# Adipose Tissue Distribution, Inflammation and Its Metabolic Consequences, Including Diabetes and Cardiovascular Disease

**DOI:** 10.3389/fcvm.2020.00022

**Published:** 2020-02-25

**Authors:** Alan Chait, Laura J. den Hartigh

**Affiliations:** Division of Metabolism, Endocrinology and Nutrition, Department of Medicine, University of Washington, Seattle, WA, United States

**Keywords:** adipokines, subcutaneous white adipose tissue, visceral white adipose tissue, brown adipose tissue, beige adipose tissue, metabolic syndrome, insulin resistance

## Abstract

Adipose tissue plays essential roles in maintaining lipid and glucose homeostasis. To date several types of adipose tissue have been identified, namely white, brown, and beige, that reside in various specific anatomical locations throughout the body. The cellular composition, secretome, and location of these adipose depots define their function in health and metabolic disease. In obesity, adipose tissue becomes dysfunctional, promoting a pro-inflammatory, hyperlipidemic and insulin resistant environment that contributes to type 2 diabetes mellitus (T2DM). Concurrently, similar features that result from adipose tissue dysfunction also promote cardiovascular disease (CVD) by mechanisms that can be augmented by T2DM. The mechanisms by which dysfunctional adipose tissue simultaneously promote T2DM and CVD, focusing on adipose tissue depot-specific adipokines, inflammatory profiles, and metabolism, will be the focus of this review. The impact that various T2DM and CVD treatment strategies have on adipose tissue function and body weight also will be discussed.

## Introduction

Obesity has now reached epidemic proportions, with over 60% of the US population classified as overweight or obese (defined by a body mass index ≥ 25 or 30 kg/m^2^, respectively) (1). The incidence of type 2 diabetes mellitus (T2DM) has also risen in parallel to the obesity epidemic, and thus is considered a major co-morbidity associated with obesity (2, 3). Recent epidemiological evidence has shown that 85% of type 2 diabetic adults are also obese (4), and it has been projected that more than 300 million people worldwide will have T2D as a consequence of obesity by 2025 (5). While much recent research has aimed to delineate the precise cause(s) of obesity-associated T2DM, the primary mechanism is believed to be insulin resistance that derives from white adipose tissue, liver, and/or skeletal muscle, accompanied by impaired insulin secretion by pancreatic β-cells (6). Furthermore, both obesity and T2DM increase the risk of cardiovascular disease (CVD), increasing morbidity and mortality by greater than 2-fold (7–10). The distribution of adipose tissue is of great importance with regards to these co-morbidities. Insulin resistance often occurs when fat accumulates in intra-abdominal depots and is associated with a constellation of CVD risk factors, in what is known as the metabolic syndrome (11). Simply measuring body weight, waist circumference, or calculating BMI does not portray a clear picture of body composition nor fat distribution. Thus, other indices have become more useful for assessing body fat distribution, such as waist-to-hip ratios, as well as methods for assessing body composition, including anthropometry, dual-energy X-ray absorptiometry (DEXA), and computed tomography (CT) scanning. A clear picture of body fat distribution in obese subjects is critical for determining how susceptible they are or will be to developing diabetes and/or cardiovascular disease. In this comprehensive review, the complex and interrelated associations between obesity, diabetes, and CVD will be explored in greater detail.

## Types of Adipose Tissue

Adipose tissue can be classified by *morphology* into white, brown, or beige subsets. In addition, white adipose tissue (WAT) can be broadly classified by *location*, largely defined as subcutaneous (located under the skin) and visceral/omental (located intra-abdominally, adjacent to internal organs). Adipose tissue is comprised of many different cell types, which coordinately secrete numerous cytokines, chemokines, and hormones. Approximately one third of the cells within adipose tissue are adipocytes, with the rest represented by fibroblasts, endothelial cells, macrophages, stromal cells, immune cells, and pre-adipocytes. In most lean, healthy individuals, WAT is confined to defined depots. But in certain conditions such as obesity and lipodystrophy, WAT mass can increase ectopically in areas that may influence the susceptibility to comorbidities such as diabetes and atherosclerosis. Such ectopic WAT areas are mostly located within the visceral cavity, and include intrahepatic (discussed in the section on Ectopic Fat below), epicardial (epiWAT, between the heart and the pericardium), perivascular (PVAT, surrounding major blood vessels), mesenteric fat (MWAT, contiguous with digestive organs in the viscera), omental fat (OWAT, an apron of fat that stretches over the intestines, liver, and stomach), and retroperitoneal fat (RWAT, surrounding the kidneys). The latter three depots (MWAT, OWAT, and RWAT) will be classified together herein as “visceral fat” (12). In addition to WAT depots, brown adipose tissue (BAT) represents a distinct type of adipose tissue that is characterized by its morphology and function, with concentrated mitochondria giving it a characteristic brown appearance. Beige fat represents a third new classification of adipose tissue, in which brown adipocytes appear within classical WAT depots. Each of these adipose depots will be discussed in more detail below.

## Adipose Tissue Distribution

### Subcutaneous Fat

Primarily localized to upper and lower body depots in humans, subcutaneous WAT is the most prominent WAT depot in lean, healthy subjects, making up ~80% of all adipose tissue (13). Thus, more than any other depot, subcutaneous WAT represents a physiological buffer for excess energy intake during times of limited energy expenditure. Subcutaneous WAT acts as a metabolic “sink” for excess lipid storage (14). When this storage capacity is exceeded, either due to an inability to generate sufficient new adipocytes (limited hyperplasia) or an inability to further expand existing adipocytes (limited hypertrophy), fat begins to accumulate ectopically in areas outside the subcutaneous WAT (see sections on Ectopic and Visceral Fat below). Additionally, subcutaneous WAT functions as an insulator to prevent heat loss, as a barrier against dermal infection, and as a protective cushion against physical external stress (15).

Subcutaneous WAT likely arises from adipocyte precursor cells that are distinct from adipocytes that arise ectopically, for example in visceral fat (16). Elegant work by Kahn et al. has demonstrated that pre-adipocytes isolated from mouse and human subcutaneous WAT expresses developmental genes that are present prior to the development of WAT in a pattern that is maintained throughout adulthood, suggesting a cell-autonomous function (16). Thus, WAT distribution has a strong heritable component (17).

The beneficial effects of subcutaneous WAT to glucose metabolism have been demonstrated in numerous ways. However, subcutaneous WAT can be further subdivided into “upper” and “lower” regions, located primarily in the trunk and gluteo-femoral regions, respectively. Upper subcutaneous WAT is often lumped together with visceral WAT, classified together as “abdominal fat.” The distinction between upper and lower subcutaneous WAT and how they contribute to metabolic health will be discussed in later sections.

### Epicardial Fat

Epicardial adipocytes share embryonic origins with mesenteric and omental adipocytes (18). epiWAT (also termed pericardial WAT) is in close proximity to the myocardium, enabling a shared microcirculation between epiWAT and certain areas of the heart (19). Due to its proximity to the heart, epiWAT is thought to be approximately twice as metabolically active as other WAT depots, with higher levels of fatty acid uptake and fatty acid release due to lipolysis (20). As a metabolically active WAT depot, epiWAT secretes several adipokines and vasoactive substances such as adiponectin, resistin, vascular endothelial growth factor (VEGF), and inflammatory cytokines and chemokines that impact the adjacent myocardium (21). In fact, due to the complete lack of a fibrous fascial layer between epiWAT and the myocardium, diffusion of fatty acids and other bioactive hormones from epiWAT to myocytes and coronary vessels is easily facilitated (22). Most humans possess a small amount of epiWAT, which provides fatty acids through lipolysis of its triglyceride stores for energy use by the heart. However, obese humans possess an enlarged epiWAT depot, which is clinically related to features of the metabolic syndrome (discussed in later sections).

### Perivascular Fat

Fat that surrounds blood vessels is termed perivascular fat (PVAT). It has now been recognized that PVAT has characteristics that resemble both BAT and WAT, and is considered to be an active participant in vascular homeostasis (23). PVAT produces many bioactive molecules that influence vascular reactivity, including adipokines (e.g., leptin, adiponectin, omentin, visfatin, resistin, and apelin), cytokines/chemokines [e.g., interleukin-6 (IL-6), tumor necrosis factor α (TNFα), and monocyte chemotactic protein-1 (MCP-1)], and vasoactive molecules (e.g., nitric oxide, prostacyclin, and angiotensin II) (24). Thus, PVAT can directly contribute to vascular tone, in addition to playing a supportive role in maintaining vessel structure. It has been suggested that PVAT in the thoracic aorta resembles BAT, while PVAT in the abdominal aorta exhibits properties of both BAT and WAT (24). Thus, if PVAT becomes dysfunctional in the setting of obesity, it can pivot from providing an atheroprotective role to promoting atherosclerosis. This concept will be evaluated further in later sections.

### Visceral Fat

Fat localized within the visceral compartment has been classified as omental, mesenteric, and retroperitoneal. Lean, healthy individuals do not have large amounts of visceral fat, which largely falls into the category of ectopic fat. Visceral fat is highly metabolically active and is constantly releasing free fatty acids (FFA) into the portal circulation. As such, visceral fat content contributes to various features of the metabolic syndrome, such as hyperinsulinemia, systemic inflammation, dyslipidemia, and atherosclerosis (25), to be discussed in more detail in later sections pertaining to obesity.

### Brown Fat

BAT is localized to distinct anatomical regions that have been well-characterized in rodents (26). By taking up circulating fatty acids, BAT functions to generate heat by uncoupling chemical energy production (ATP) via oxidative phosphorylation into heat production (non-shivering thermogenesis), thereby contributing to the clearance of plasma triglycerides and the mitigation of ectopic lipid storage (27). While originally believed to be a depot exclusive to hibernating and small mammals, and present to some degree in human infants, adult humans have recently been shown to have functional and inducible levels of BAT that respond to cold and sympathetic nervous system activation (28–30). Such BAT represents between 1 and 2% of total fat stores in humans, and is localized primarily in the cervical, axillary, and paraspinal regions (26, 31, 32). Similarly to WAT, BAT synthesizes and secretes “batokines” such as fibroblast growth factors (FGFs) including FGF21, neuregulin 4, VEGF, and cytokines such as IL-6 (33). Given the relatively small amount of BAT present in humans, the endocrine potential of batokines is relatively unknown, but it is clear that factors secreted from BAT exert paracrine and autocrine functions. While the relative BAT mass in humans and rodents is small compared to other adipose depots, its relative contribution to metabolic health may be higher.

In rodents and other small mammals, the primary BAT depots are located in the interscapular space and supraclavicular regions, among many others (26, 34). With prolonged stimulation, i.e., cold exposure, the size and activity of these BAT depots will increase, a term called BAT recruitment. BAT recruitment is associated with enhanced proliferation and differentiation of BAT precursor cells.

### Beige Fat

In addition to WAT and BAT, a third fat type has been described, termed “browned,” “beige,” or “brite” (brown-in-white) fat. As the name suggests, beige fat has been described as the presence of brown adipocytes within classic WAT depots. While beige fat shares some features of classical BAT such as systemic triglyceride-lowering, beige fat is thought to be physiologically distinct from BAT, with differential expression of certain genes involved in metabolism, inflammation, and transcription (35, 36). Moreover, human BAT exhibits similar morphology and function as both rodent BAT and beige tissue (30, 37–39), complicating comparisons between the two species. In rodents, subcutaneous WAT is the most susceptible depot to browning, while in humans it is visceral WAT (40). It is generally believed that the majority of WAT depots can develop browning under particular conditions, but more work is needed in this area. There is a growing list of physiological stressors that can promote the browning of WAT, including cold exposure, exercise, bariatric surgery, cancer cachexia, severe burns, as well as pharmacological and dietary components such as conjugated linoleic acid, short-chain fatty acids, capsaicin, non-caffeinated green tea extract, thiazolidinediones (TZDs), and β-adrenergic receptors (41–52).

There is some debate regarding the origins of beige adipocytes, as well as their impact on energy homeostasis. Beige adipocytes may arise from *de novo* adipogenesis from specific progenitor cells when initially stimulated by cold exposure (36, 53), but then may lie “dormant” until stimulated again (54). This theory suggests that dormant beige adipocytes can become quickly and readily activated when needed, reminiscent of an immune response. This newly defined relative flux between “dormant” and “active” beige cells may be what has been previously termed “transdifferentiation” of white-to-beige adipocytes (54). Beige adipocytes were initially thought to arise from transdifferentiation from white adipocytes, with the ability to de-differentiate back into white adipocytes (55, 56). Additional studies *in vitro* suggest that this is likely not the case (57). The identity of committed beige adipocyte precursors has not been fully elucidated, but there is evidence from isolated WAT stromal cells that beige adipocyte precursors are distinct from white adipocyte precursors (36, 39, 58). It has been suggested that strategies that increase the number of beige adipocytes in mouse WAT also protect them from diet-induced obesity (59–63).

## Normal Adipose Tissue Function

### White Adipose Tissue: Energy Storage and Distribution

Adipose tissue is an essential organ for the regulation of energy homeostasis. Primarily tasked with storing excess energy as triglycerides, adipocytes undergo hyperplasia to increase the number of adipocytes and hypertrophy to increase the size of each adipocyte, allowing adipose tissue to expand in times of nutrient excess. As needed, i.e., during fasting and exercise, triglycerides stored in adipose tissue are mobilized to provide fatty acids for energy utilization by the rest of the body. Stored triglycerides are therefore in a constant state of flux, whereby energy storage and energy mobilization are determined largely by hormonal fluctuations. Thus, adipose tissue functions as an energy balance “hub” that integrates and services the energy requirements of diverse organ systems, such as the liver, skeletal and heart muscle, pancreas, and brain (64).

In healthy lean individuals, the majority of adipose tissue resides in subcutaneous depots, where it serves a thermoregulatory function, and from which stored triglycerides can be readily mobilized when needed (65). Conditions that favor adipose tissue expansion, if endured chronically, will eventually exceed the storage capacity of defined adipose tissue depots, leading to the ectopic deposition of triglycerides in other tissues, including intra-abdominal depots (discussed in more detail in later sections).

### Non-shivering Thermogenesis

BAT plays an important role in thermoregulation in mammals, including adult humans (66). BAT tissue is rich in mitochondria and uniquely expresses uncoupling protein-1 (UCP-1), which enables heat production by uncoupling ATP synthesis. BAT-mediated thermogenesis has garnered substantial attention recently, as increasing BAT mass or activity could be an effective strategy to combat obesity. While the primary function of WAT is to manage energy storage, brown adipocytes efficiently burn fatty acids released from WAT during adaptive thermogenesis (67). BAT plays an active role in metabolism in animals and humans (28); therefore, strategies that increase BAT mass and/or activity could promote fat loss in obese populations. In addition, beige fat could also contribute to fat catabolism, potentially reducing WAT stores. Human brown adipogenesis occurs in response to chronic or repeated cold stimulation, or in response to pharmacologic compounds such as beta adrenergic receptor (β-AR) agonists (68, 69). However, these browning-inducing methods mediated by the sympathetic nervous system are not practical as a weight loss strategy for several reasons: (1) the browning effects of cold exposure are rapidly reversible, (2) repeated cold exposure is too time- and energy-consuming to be a practical therapeutic, and (3) β-ARs promote adverse cardiometabolic events. Therefore, mechanisms of WAT browning that are long lasting and act independently from the sympathetic nervous system are highly sought after. A new mechanism of WAT browning that does not involve the sympathetic nervous system (SNS) has recently been described. Adipose tissue resident macrophages can secrete norepinephrine (NE), the neurotransmitter that is also secreted by sympathetic neurons to activate BAT and WAT browning (70). Several follow up studies have suggested that eosinophils, type 2 cytokines, and alternatively activated macrophages play critical roles in supporting WAT browning with concomitant increased energy expenditure and weight loss (71–79). However, the notion that immune cells can influence WAT browning has recently been challenged, using different murine and *in vitro* approaches (80). As such, there is some discordance regarding the role of macrophages in WAT browning, necessitating further studies.

### Secretion of Hormones and Adipokines

Originally classified as a simple energy storage organ, adipose tissue is now known to function as a major endocrine system that secretes adipokines, growth factors, cytokines, and chemokines (81). The secretion pattern of adipokines appears to vary by adipose tissue depot and is dependent on the energy status of the adipose depot, leading to variable paracrine/autocrine effects of adipokines within particular depots. Adipokines are important mediators of various metabolic processes such as fatty acid oxidation, *de-novo* lipogenesis, gluconeogenesis, glucose uptake, insulin signaling, and energy expenditure in metabolically active tissues such as the liver, skeletal muscle, and brain (81). The various adipokines secreted from adipose tissue and their functions will be described in more detail below. The discussion will be limited to adipokines that are known to be produced to a large extent by adipocytes, in addition to other cell types within adipose tissue such as immune cells.

#### Leptin

Discovered in 1994, leptin is a peptide hormone that is expressed exclusively by adipocytes and is essential for body weight regulation. Leptin, adiponectin, and omentin (the latter two will be described below) are the only generally accepted adipokines with true endocrine function, meaning they are released from adipose tissue and exert effects on distant target organs. Leptin is encoded by the obesity gene (*ob*). Leptin-deficient (*ob*/*ob*) mice become spontaneously obese due to unrestricted food intake, highlighting the importance of this adipokine in suppressing appetite through the central nervous system (82). Rodents and humans that lack either leptin or the leptin receptor (LEPR) are not only extremely obese, but are also hyperglycemic and extremely insulin resistant (83). In lean and obese animals and humans, circulating leptin levels positively correlate with adiposity (84). Prolonged fasting is associated with a sharp drop in plasma leptin levels, which drives food intake (85). While leptin is expressed in all adipose depots, including BAT, its expression is highest in subcutaneous WAT (86).

#### Adiponectin

As one of the first adipokines discovered in the mid-1990s (87–90), adiponectin is a well-described insulin-sensitizing hormone that impacts a wide range of tissues. Adiponectin is a distinctly unique adipokine, as its expression and circulating levels are inversely proportional to adiposity levels, in stark contrast to leptin. Adiponectin expression levels vary between sexes, with higher levels observed in females than males (91–93), and between adipose tissue depots, with higher expression in subcutaneous than visceral WAT (94, 95). The insulin sensitivity-promoting properties of adiponectin are well-known, and are exemplified by the development of insulin resistance in adiponectin-deficient mice (96), and the preservation of insulin sensitivity in adiponectin-overexpressing mice (97). Adiponectin signals through two related receptors, ADIPOR1 and ADIPOR2, followed by docking of the adaptor protein APPL1 (98). The resulting signaling pathway, mediated through peroxisome proliferator-activated receptor alpha (PPARα), leads to metabolic improvements involving decreased hepatic gluconeogenesis, increased liver and skeletal muscle fatty acid oxidation, increased glucose uptake in skeletal muscle and WAT, and decreased WAT inflammation (99). Thus, adiponectin receptors are highly expressed in skeletal muscle, liver, and adipose tissue. In addition, adiponectin receptors are expressed in the pancreas, where adiponectin functions to mitigate β-cell loss by neutralizing inflammatory and lipotoxic ceramides and diacylglycerols (100). In addition to β-cells, adiponectin has also been shown to exhibit strong anti-inflammatory effects on other cell types such as macrophages and fibrogenic cells (99, 101, 102). Taken together, adiponectin plays a protective role in mitigating features of the metabolic syndrome.

#### Resistin

Resistin is a polypeptide that is secreted by obese adipose tissue. It was originally described as an adipocyte-specific hormone, but it is now thought to originate from macrophages residing in inflamed adipose tissue in mice (103) and from circulating monocytes and tissue macrophages in humans (104, 105). Human resistin is only 59% homologous to mouse resistin (106), which has raised some controversy over the pathogenic role of resistin, and limits comparisons between animal models and human disease (107). Resistin is so named due to its ability to “resist,” or interfere with insulin action (108), based on initial studies in mouse models. Evidence for this comes from an initial study in which it was observed that plasma resistin levels are elevated in a diet-induced obese mouse model, that blocking resistin action using a neutralizing antibody improves insulin sensitivity, and that recombinant resistin administration to healthy mice promotes insulin resistance (108). These initial studies led to the suggestion that resistin plays an important role in modulating insulin resistance in the context of obesity, and it has been shown to correlate with insulin resistance in mice and humans (109). Plasma resistin levels have been shown to be increased in obese animal models and humans (110–113) and to decrease with weight loss in humans (114). Conversely, some studies have shown that adipose tissue-derived resistin is suppressed in obesity (115–117), inciting the controversy over what role resistin plays in obesity that persists today. Evidence suggests that visceral fat is the largest contributor to circulating resistin levels (113), supporting the case for an association between resistin and insulin resistance. Moreover, resistin is believed to be an active participant in propagating inflammatory responses. Resistin can upregulate inflammatory cytokines such as TNFα and IL-6 in monocytes and macrophages in a nuclear factor kappa-B (NFκB)-dependent manner (118), and is positively associated with circulating inflammatory markers such as C-reactive protein (CRP) and TNFα (107). Thus, while resistin is an established adipokine and has been shown in some cases to be associated with adverse health conditions such as obesity and insulin resistance, a clear role for resistin is still under active investigation.

#### Omentin

Initially described as an adipokine secreted from omental WAT (119), it is now generally accepted that omentin is also expressed in other WAT depots such as epicardial fat, and that it derives specifically from the stromal vascular fraction of WAT (119, 120). Omentin is a true endocrine hormone that circulates in the blood (121, 122). Omentin levels are reduced in subjects with obesity (123) and T2DM (124, 125), leading investigators to speculate that omentin may be involved in glucose homeostasis. Indeed, studies using *in vitro* models showed that omentin enhances insulin-stimulated glucose uptake in human adipocytes by activating Akt signaling pathways (119), and studies in humans show a significant negative correlation between serum omentin levels as well as adipose omentin mRNA levels with insulin resistance (124, 126, 127). Omentin levels have been shown to gradually increase in response to weight loss (128, 129). Additional studies suggest that omentin has anti-inflammatory properties. Omentin blunts cytokine expression in endothelial cells (130), vascular smooth muscle cells (131, 132), macrophages (133), cardiomyocytes (134), and adipose tissue itself (135), and is negatively associated with systemic inflammatory markers such as TNF and IL-6 (136). Thus, omentin is considered to be a biomarker for metabolic health that may function to blunt obesity-related cytokine effects (137).

#### Fibroblast Growth Factor 21 (FGF21)

FGF21 is an endocrine hormone that is involved in the regulation of lipid, glucose, and energy homeostasis (138). FGF21 has received a lot of attention for its insulin-sensitizing and weight loss-inducing effects when administered pharmacologically (139). The liver is the primary source of circulating FGF21, induced by metabolically stressful conditions such as fasting, a ketogenic diet, protein restriction, and bariatric surgery (140), while the brain and adipose tissue are primary FGF21 targets (141, 142). Other tissues are known to also secrete FGF21, including the pancreas and skeletal muscle (143, 144). However, under certain metabolic conditions such as obesity, WAT and BAT may also produce FGF21 (145). This is supported by several studies showing that BMI and adiposity positively correlate with circulating FGF21 levels in mice and humans (145–149). It is clear that FGF21 levels become elevated as obesity develops in mice and humans, and are positively correlated with BMI, adiposity, and FGF21 expression levels in adipose tissue (145–149). While many studies have shown that adipose tissue expresses FGF21 in rodents (145, 150–154), there is still some debate about whether FGF21 is readily expressed in human adipose tissue. There are a handful of studies that suggest that adipose tissue *FGF21* mRNA expression is below detection levels (155) or not expressed by adipose tissue (156). However, numerous additional studies have found detectable *FGF21* mRNA expression in visceral WAT (157, 158), subcutaneous WAT (145, 157, 158), epicardial WAT (159), cervical adipose tissue (160, 161), and PVAT (162, 163), with the latter two depots containing both WAT and BAT. FGF21 protein has also been detected in adipose tissue by Western blot and immunohistochemistry (162). It is not clear why some but not all groups have been able to detect *FGF21* expression in human adipose tissue, but could depend on the metabolic and/or nutritional status of the subjects sampled (e.g., whether subjects were fasting or fed).

Some studies suggest that adipose-derived FGF21 is a marker of metabolic stress, as it has been shown to correlate with features of the metabolic syndrome (145, 164, 165). Regardless, a clearly-defined function of adipose-derived FGF21 has not yet been established, nor whether adipose-derived FGF21 promotes primarily local effects or contributes to the circulating FGF21 pool under particular metabolic conditions. Elegant studies using tissue-specific Fgf21 KO mice show that adipocyte-derived Fgf21 is not involved in obesity-associated insulin resistance, and that adipose-derived Fgf21 doesn't circulate, instead acting in a paracrine fashion (140). However, the mice used in that study were fasted for 24 h, introducing a metabolic stress that would likely only induce liver-derived Fgf21 that may have masked any contribution from adipose-derived Fgf21. In later studies, a thermogenic role for adipose-derived Fgf21 has been described, in which the browning of WAT was shown to require adipocyte-Fgf21 (141, 166). Thus, it is possible that hepatic- and adipose-derived FGF21 are induced by different stimuli, and that more studies are required to conclusively define a role for adipose-derived FGF21.

## Obesity

Obesity results when energy intake chronically exceeds energy expenditure. Many factors are involved, including genetic, epigenetic, hormonal, and lifestyle factors that are beyond the scope of this review. Adipocyte number is believed to be tightly regulated and determined during childhood (167). However, during the development of obesity, adipose tissue can expand by either hypertrophy (an increase in adipocyte size) or hyperplasia (an increase in adipocyte number due to the recruitment of new adipocytes). Obesity is characterized by dysfunctional adipose tissue, in which adipocytes initially become hypertrophic during periods of caloric excess and secrete adipokines that result in the recruitment of additional pre-adipocytes, which differentiate into mature adipocytes as compensatory protection against some of the adverse metabolic consequences of obesity (168). This concept is supported by observations in AdipoChaser mice, a model for tracking adipogenesis (169). AdipoChaser mice fed a high fat diet display evidence of hypertrophy of visceral WAT within 1 month, while hyperplasia occurs after 2 months. Importantly, subcutaneous WAT does not undergo hyperplasia, and hypertrophy lags behind the visceral compartment, with evidence of subcutaneous WAT hypertrophy after 2 months of high fat feeding (170). However, when the capacity for adipocyte recruitment and hypertrophy is overwhelmed, fat accumulates in ectopic sites such as visceral depots, the liver, skeletal muscle, and pancreatic beta cells. These changes are accompanied by inflammation, insulin resistance and other features of the metabolic syndrome, and have been termed metabolically unhealthy obesity (MUHO) (171, 172). In contrast to MUHO, some people accumulate fat mainly in subcutaneous depots, a condition that has been termed metabolically healthy obesity (MHO). MHO is not accompanied to any great extent by insulin resistance, adipose tissue and systemic inflammation, and other features of the metabolic syndrome such as dyslipidemia and hypertension (173–176). Thus, the distribution of fat accumulation is a major determinant of metabolic complications associated with obesity, which can increase the risk of CVD. Various features that contribute to dysfunctional WAT in obesity will be discussed in the sections that follow.

### Metabolically Healthy Obesity (MHO)

A sub-group of obese individuals remain insulin-sensitive, and exhibit normal metabolic and hormonal profiles despite having a BMI that would characterize them as obese (177, 178). Such individuals have been classified as having “metabolically healthy obesity” (MHO), and appear to be distinct from those with “metabolically unhealthy obesity” (MUHO) in that they remain insulin sensitive and do not have much adipose tissue inflammation or other features of the metabolic syndrome (179, 180). Therefore, MHO individuals have a lower risk for developing T2DM and cardiovascular disease (174). MHO is sometimes defined as having 2 or less features of the metabolic syndrome or based on homeostatic model assessment of insulin resistance (HOMA-IR) measures, but consensus on a precise definition does not exist (176). Thus, some individuals classified as having MHO rather fall somewhere between metabolically healthy and unhealthy. Moreover, individuals with so-called MHO can progress to develop features of the metabolic syndrome with time (181–184). Because CVD outcomes in general relate to the number of metabolic abnormalities present in individuals with MUHO (185–188), there is less CVD in individuals with MHO than those with the metabolic syndrome. In addition, while MHO individuals are so defined due to a healthier cardiometabolic profile than those with MUHO, the true clinical benefits of MHO remain in question, as the cardiometabolic profile and insulin sensitivity of MHO individuals typically does not improve significantly with weight loss (179, 189–192). Nevertheless, evidence from animal models and cultured adipocytes do suggest that the preservation of the capacity for subcutaneous WAT expansion mitigates extensive visceral and hepatic fat accumulation, potentially driving the MHO phenotype (76, 97, 193).

### Metabolically Unhealthy Obesity (MUHO)

#### Visceral Adiposity and the Metabolic Syndrome

**Figure 1:**
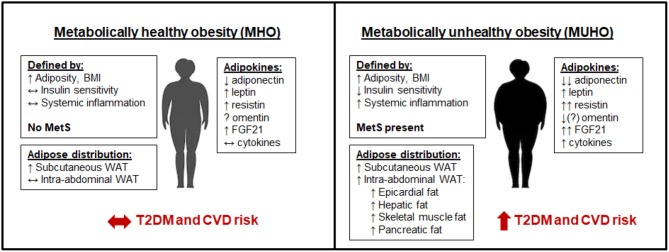
Metabolically healthy obesity (MHO) vs. metabolically unhealthy obesity (MUHO). In comparison with lean metabolically healthy subjects, those with MHO have increased adiposity and BMI, but with reduced systemic inflammation and retained insulin sensitivity, thus defining them as not having metabolic syndrome (MetS). MHO subjects have elevated subcutaneous white adipose tissue (WAT) levels, without excessive accumulation of visceral fat. Their adipokine profile is similar to lean subjects, but with increased leptin, resistin, and FGF21, and decreased adiponectin, which limits their risk of developing type 2 diabetes mellitus (T2DM) and cardiovascular disease (CVD) in the short term. By contrast, those with MUHO exhibit elevated insulin resistance and systemic inflammation in addition to increased adiposity and BMI over lean controls, contributing to MetS. MUHO individuals have excess subcutaneous and intra-abdominal adipose tissue, with increased hepatic fat and fat distributed amongst other visceral organs. This leads to a dysfunctional adipokine profile, characterized by reduced adiponectin and omentin, with further elevated leptin, resistin, FGF21, and cytokines when compared to lean controls. Thus, MUHO subjects are at risk for developing T2DM and CVD.

Other obese individuals tend to accumulate fat mainly intra-abdominally in visceral depots, which is also known as central obesity. Visceral adiposity is associated with insulin resistance, a predisposition to diabetes, local and systemic inflammation, dyslipidemia [characterized by hypertriglyceridemia, a preponderance of small, dense low-density lipoprotein (LDL) particles and reduced high-density lipoprotein (HDL)-cholesterol levels], insulin resistance, dysglycemia [a broad term that refers to an abnormality in blood sugar stability], adipose tissue and systemic inflammation, hypertension, a thrombogenic profile and non-alcoholic fatty liver disease (NAFLD) (194). This constellation of CVD risk factors associated with visceral obesity is widely known as the metabolic syndrome and is a hallmark of MUHO, illustrated in Figure 1. Visceral obesity and the metabolic syndrome are associated with an increased risk of developing CVD, which is exacerbated when overt diabetes develops as a result of insulin secretion failing to adequately compensate for insulin resistance. Interestingly, even normal weight individuals who accumulate fat intra-abdominally have these metabolic abnormalities (195, 196), including an increased risk of CVD. Asians and Asian-Americans are particularly prone to accumulate intra-abdominal fat and have features of the metabolic syndrome despite having normal weights and BMI values by Western standards (196), raising the question of whether different normal values should apply to individuals of Asian ancestry. Moreover, this raises the question of the validity of body weight or body mass index (BMI -weight in kg/height in m^2^) as an index of obesity or adiposity, since these measures do not differentiate the 2 major types of obesity. Measures such as waist circumference, waist/hip ratio and weight to height ratio have been used. These indexes are notable for their inclusion of upper subcutaneous WAT, which some consider to contribute as much, if not more, to metabolic syndrome than visceral WAT alone (197). CT scanning at the level of the umbilicus has been found to be useful but is expensive and not practical other than for research purposes at present. Lower body subcutaneous WAT does not correlate with risk factors for the metabolic syndrome, potentially due to a slower FFA turnover, higher levels of adipocyte hyperplasia, and lower levels of inflammation (198–201).

Notable differences in the adipokine profile between MHO and MUHO subjects have been reported, which could contribute to their respective risks for T2DM and CVD. Leptin has been shown to be higher in MUHO than MHO obese Chinese children in one study (202), but was not found to differ between adult groups in several other studies (203–205). By contrast, adiponectin has consistently been shown to be higher in subjects with MHO than in those with MUHO, despite both populations having lower adiponectin than metabolically healthy lean controls (203, 205–209). Resistin and FGF21 levels tend to be highest in the MUHO population (148, 208). Data on whether omentin levels differ between MHO and MUHO has been inconsistent, with one study suggesting that MUHO subjects have higher omentin levels than MHO subjects (210), and other suggesting the opposite, that omentin levels are negatively correlated with the metabolic syndrome (122, 211). Cytokines such as TNFα and IL-6 as well as the chemokines SAA and MCP-1 have been shown to be elevated in MUHO (208). These adipokine differences between subjects with MHO and MUHO are depicted in Figure 1.

#### White Adipose Tissue Inflammation

##### Macrophages and inflammation

Adipose tissue expansion in obesity is accompanied by inflammatory changes within adipose tissue, contributing to chronic low-grade systemic inflammation that is characterized as mildly elevated levels of circulating cytokines, chemokines, and acute phase reactants. In mice fed a high fat diet, obesity is associated with the induction of a large number of inflammatory pathways, constituting as many as 59% of total pathways that are differentially regulated (212). Expansion of adipose tissue depots during weight gain is accompanied by an infiltration of new inflammatory cells, the major one initially being macrophages. Reported to represent ~5–10% of total cells within lean adipose tissue, macrophages in obese adipose tissue represent up to 60% of all cells present (213). These pro-inflammatory cells are recruited in response to chemokines such as monocyte chemotactic protein-1 (MCP-1) produced by hypertrophic adipocytes (213, 214). Studies in mice have demonstrated that most macrophages in obese adipose tissue are derived from circulating monocytes (213), although a small percentage appear to derive from proliferation of resident tissue macrophages (215). Resident macrophages that are present in normal adipose tissue express markers of “alternatively activated,” or M2 macrophages such as the mannose receptor (CD206), macrophage galactose type C-type Lectin/CD301a/CLEC10A (MGL1), and arginase-1 (ARG1). These anti-inflammatory macrophages are believed to be responsible for maintaining tissue homeostasis (216). It remains unclear whether the derivation of adipose tissue macrophages is the same in human obesity.

Macrophage accumulation occurs to a greater extent in visceral than in subcutaneous adipose depots in both rodents and humans (217–220). Macrophages are seen in crown-like clusters, where they are thought to represent an immune response to dead and dying adipocytes (219). These recruited macrophages demonstrate a phenotypic switch from being anti- to pro-inflammatory, and develop some features similar to “classically activated,” or “metabolically-activated” macrophages (MMe) (221–223). However, use of genetic markers show that these cells have significant differences from classical M1 macrophages and alternate nomenclatures have been suggested for these pro-inflammatory cells. Morris and Lumeng have divided adipose tissue macrophages into several populations based on cell surface markers and expression profiling (224). Using a proteomics approach, Kratz et al. showed that markers of classical activation were absent on ATMs from obese humans. Stimulation of macrophages with glucose, insulin, and palmitate resulted in the production of a “metabolically activated” MMe phenotype distinct from classical activation. Such markers of metabolic activation were expressed by pro-inflammatory macrophages in adipose tissue from obese humans and mice and correlated with the extent of adiposity (225).

##### Other immune cells

In addition to macrophages, T-cells also are present in normal adipose tissue and demonstrate phenotypic change during weight gain. Both CD4+ and CD8+ T cells are found in adipose tissue and are increased in the obese state. Th2 cytokines (e.g., IL-4 and IL-13) are responsible for generating “alternatively activated” (M2) macrophages in lean adipose tissue. With weight gain in mice there is a shift away from a predominance of TH2 T cells present in lean adipose tissue and toward more TH1 and cytotoxic T cells as well as a reduction in regulatory T cells (Tregs) (226). Interferon γ (IFNγ)–expressing Th1 polarized T cells appear to promote adipose tissue inflammation and increased IFN-γ activity has been reported in adipose tissue in both mice and humans (227, 228). T-cell activation involves peptide antigen presentation via major histocompatibility complex (MHC) class II (CD4+) or MHC class I (CD8+). A subset of T cells called natural killer T (NKT) cells respond to lipid or glycolipid antigens (229–231). The number of invariant NKT (iNKT) numbers has been observed to be reduced in adipose tissue and livers from obese mice and humans (232–235). B-cells and mast cells also are increased in adipose tissue in the obese state (227, 236, 237). Use of specific cell surface markers has also demonstrated the presence of dendritic cells in adipose tissue, and studies indicate that dendritic cells are independent contributors to adipose tissue inflammation during obesity (238, 239).

##### Chronic inflammation in obesity

Adipose tissue inflammation in obesity differs from typical inflammatory responses employed in host defense in that it is chronic, sterile, low grade, and affects the metabolic control of nutrient flow in adipose tissue, liver, muscle and pancreas, and has been termed “meta-inflammation.” One way it affects nutrient flow is by causing insulin resistance. There is good evidence to support the notion that the systemic inflammation that is associated with obesity and contributes to insulin resistance begins with adipose tissue inflammation. The regulation of hepatic C-reactive protein (CRP) and serum amyloid A (SAA) is likely in response to IL-6 secretion from visceral adipose tissue that directly targets the liver via the portal circulation (240–244). CRP is a prominent biomarker for insulin resistance and CVD (245–247), and SAA antagonizes insulin action in adipocytes, thus contributing to systemic insulin resistance (248). SAA also has been associated with CVD in some rodent and human models (218, 249–253). In summary, the discovery of elevated secretion of inflammatory cytokines by obese adipose tissue provides evidence that obesity directly mediates systemic inflammation, which contributes to insulin resistance and CVD (discussed further in later sections).

#### Cytokines and Chemokines

Obesity is associated with elevated circulating levels of IL-6 and TNFα, which are subsequently decreased with weight loss (254, 255). Adipose tissue is a major source of these cytokines (256) as well as the chemokine MCP-1, which is important for recruitment of inflammatory cells such as macrophages to expanding adipose tissue (257). While such inflammatory mediators that originate from adipose tissue could technically be classified as adipokines, they are also produced by the majority of cell types in the body and will therefore be described in further detail in this section. It should be noted that cytokine and chemokine production is limited in lean adipose tissue and in subjects with MHO. Many cell types synthesize and secrete these cytokines and chemokines, including several that make up the adipose tissue milieu such as monocytes, macrophages, dendritic cells, B cells, and T cells. As such, they play a prominent role in adipose tissue pathophysiology associated with obesity.

##### IL-6

Much research has been devoted to the role that adipose-derived IL-6 plays in the etiology of obesity. The expansion of adipose tissue is accompanied by excessive adipocyte lipolysis and subsequently elevated FFA levels, which promotes adipocyte IL-6 secretion (258, 259). Omental fat produces 2 to 3-fold higher levels of IL-6 than subcutaneous fat (260), providing a potential mechanism for the higher contribution of omental WAT to insulin resistance (261). Most studies *in vitro* and in mice suggest that adipose-derived IL-6 promotes hepatic insulin resistance and glucose intolerance (259, 262, 263), while some indicate that in certain contexts IL-6 signaling in WAT and liver may be protective against metabolic disease (264, 265). For example, mice with genetic disruption of the IL-6 receptor specifically in the liver exhibit exacerbated hepatic inflammation and impaired glucose tolerance (264), suggesting that IL-6 may also function to limit hepatic inflammation. Thus, the context in which IL-6 signaling is studied is critically important for the interpretation of its function.

##### TNFα

In addition to its secretion from inflammatory cells such as monocytes and macrophages, TNFα was first described as an adipokine in 1993 (266). As with IL-6, TNFα levels positively correlate with adiposity, BMI, insulin levels, and insulin resistance (267, 268). While adipocytes themselves can secrete TNFα, the majority of TNFα secreted from adipose tissue is derived from immune cells in the stromal vascular fraction, and that obesity-associated increases in TNFα largely reflect the infiltration of pro-inflammatory macrophages within expending adipose tissue (213). One mechanism by which adipose-derived TNFα may promote insulin resistance is by directly activating hormone sensitive lipase (HSL), thereby increasing FFA release from adipocytes which promotes insulin resistance in the liver and skeletal muscle (269). Another mechanism is via autocrine activation of insulin receptor substrate-1 (IRS-1), which prevents insulin from interacting with its receptor (270).

##### MCP-1

Monocyte chemotactic protein-1 (MCP-1) is a potent chemotactic factor that promotes monocyte and macrophage recruitment into sites of inflammation during tissue injury and infection. It is secreted by adipocytes during the development of obesity and leads to infiltration of monocytes, which differentiate to become adipose tissue macrophages. The macrophages in turn secrete additional MCP-1 leading to further recruitment of inflammatory cells (271, 272). Body mass index and adiposity strongly correlate with adipose *CCL2* (the gene encoding MCP-1) expression levels, and MCP-1 decreases following weight loss in humans (273). *Ob*/*ob* mice, a commonly used mouse strain that spontaneously develops obesity due to leptin deficiency-induced hyperphasia, as well as diet-induced obese mice, display elevated levels of plasma Mcp-1 and *Ccl2* adipose tissue expression (213, 274, 275). In addition, mice engineered to express elevated levels of *Ccl2* specifically from adipocytes exhibit increased macrophage recruitment into adipose tissue, and subsequently increased insulin resistance, effects that were not observed in diet-induced obese mice that were deficient in *Ccl2* (274). Potential mechanisms by which adipose-derived MCP-1 could increase insulin resistance include changes in liver mRNA expression of genes involved in lipid and glucose metabolism in response to elevated FFA (274), or more likely due to increased recruitment of macrophages into adipose tissue (described in the section on “Obesity and insulin resistance”). Evidence suggests that human visceral WAT secretes higher levels of MCP-1 than subcutaneous WAT (276). These studies and others have prompted the suggestion that MCP-1 could be a viable therapeutic target for the treatment of obesity and associated insulin resistance.

##### Serum amyloid A (SAA)

While well-described as an acute phase protein secreted by the liver in response to pro-inflammatory cytokines, SAA is also expressed in adipocytes and macrophages and correlates with adiposity (244, 277–281). There are 4 subtypes of SAA: SAA1–4. SAA1 and SAA2 are highly upregulated in response to inflammation, while SAA4 is largely constitutively expressed. SAA3 is a pseudogene in humans, replaced by SAA1 and SAA2 in extra-hepatic tissues. While the best defined cell source of SAA1 and SAA2 is hepatocytes, SAA1 and SAA2 are also expressed from adipocytes and macrophages under inflammatory conditions in metabolic diseases such as obesity, insulin resistance, and cardiovascular disease (250). SAA3 expression is increased during hypertrophy of cultured mouse adipocytes (214) and in gonadal fat in obese mice (218, 282). Inducible forms of SAA also are expressed in both subcutaneous (277) and omental WAT (283) from obese humans. Thus, the increased adipocyte size and number that accompanies obesity is also associated with elevated adipose tissue-derived SAA levels, likely in part due to increased hepatic secretion in response to cytokines produced in adipose tissue.

#### Ectopic Fat

In obesity, white adipose tissue may become dysfunctional and unable to properly expand to store excess ingested energy, triggering storage of triglycerides in sites where the primary function is not fat storage. Ectopic fat that is localized to major glucose regulatory organs such as the liver, skeletal muscle, and pancreas is commonly regarded as being “lipotoxic,” since this ectopic fat can interfere with normal insulin signaling and promote insulin resistance and increase the risk for T2D (284, 285). Excessive amounts of visceral fat also is considered to be a form of ectopic fat, and as noted earlier, is associated with features of the metabolic syndrome and an increased risk of T2DM and cardiovascular complications (286). In animal models as well as in humans, it has been shown that the accumulation of lipotoxic diacylglycerols (DAGs) and ceramide, as occurs with visceral obesity, leads to impaired insulin signaling and reduced glucose uptake in skeletal muscle and liver (287–290). More specific mechanisms by which ectopic fat accumulation in particular tissues promotes insulin resistance will be explained in the following sections.

##### Hepatic lipid accumulation and inflammation

Several studies have reported an inverse relationship between hepatic lipid content and whole-body insulin sensitivity (291–293). The liver is a major target for the excessively produced inflammatory cytokines and FFAs released from obese WAT (294) (see later). It has been estimated that nearly 60% of ectopic hepatic triglycerides in obese NAFLD patients derive from FFA released from adipose tissue (295). FFA-derived triglycerides accumulate in the cytoplasm of hepatocytes in the form of lipid droplets. While the lipid droplets may not be lipotoxic *per se*, various intermediate lipid moieties generated during triglyceride synthesis (e.g., DAGs and ceramide) have been shown to promote lipotoxicity and enhance hepatic insulin resistance (296), likely by inhibiting insulin signaling pathways (297, 298). Selective upregulation of ceramide degradation pathways in the liver has been shown to reverse hepatic lipid accumulation and improve glucose tolerance in diet-induced obese mice (299). Moreover, obesity-associated reductions in adiponectin have also been shown to contribute to hepatic steatosis, presumably by blunting hepatic fatty acid oxidation, a process regulated by adiponectin (300–302).

It also has been suggested that adipose tissue inflammation contributes to hepatic lipid accumulation. Kanda et al. showed that overexpressing *Ccl2* from adipocytes in mice led to macrophage accumulation in adipose tissue and subsequent hepatic steatosis and hepatic insulin resistance, without an obese phenotype (274). Similarly, mice in which *Ccl2* had been deleted showed resistance to high fat diet-induced insulin resistance and hepatic steatosis, an effect that was accompanied by reduced expression of TNFα in adipose tissue (274). Additional evidence to support the notion that adipose tissue inflammation promotes hepatic steatosis derives from studies showing that adipose-derived cytokines promote lipolysis of WAT stores (303, 304), thus increasing circulating FFA levels.

Kupffer cells are liver-resident macrophages, and reportedly comprise 80–90% of all tissue-resident macrophages in the body (305). In the healthy liver, the role of Kupffer cells is to phagocytose pathogens and toxins and to maintain tissue homeostasis and repair, akin to an M2 macrophage (306, 307). In contrast with adipose tissue macrophages, hepatic Kupffer cell numbers do not increase with adiposity, but instead become “activated,” akin to M1 or MMe macrophages (308). The primary stimuli for Kupffer cell activation likely derive from dysfunctional adipose tissue, including FFA, cytokines, and adipokines (309). Adipokine imbalance such as the hypoadiponectinemia that results from visceral adipose tissue expansion fails to suppress hepatic inflammation and oxidative stress, contributing to Kupffer cell activation. Thus, signals from dysfunctional obese adipose tissue propagate hepatic inflammation by activating resident Kupffer cells, which then themselves secrete pro-inflammatory cytokines, further amplifying systemic inflammation (310).

##### Ectopic fat in skeletal muscle

Lipids also can be stored within skeletal muscle when the capacity for fat storage by WAT is exceeded (311). Lipids can be stored either between muscle fibers (as adipocytes, or extramyocellular lipids), or within muscle cells (cytosolic triglycerides, or intramyocellular lipids) (312). Pre-adipocytes have been identified within skeletal muscle, providing evidence that distinct adipocyte cells may reside between skeletal muscle fibers (313). There is an association between ectopic skeletal muscle fat and insulin resistance that is largely dependent on BMI, but this association persists when BMI is statistically accounted for (314–316). It remains to be determined whether skeletal muscle fat is simply a marker of metabolic dysfunction or if it plays an active role in mediating insulin resistance. Ectopic skeletal muscle fat, as with ectopic fat in other areas, has the potential to impair insulin action in skeletal muscle through the inhibition of insulin signaling by lipotoxic DAGs and ceramide (317, 318). Several large clinical trials including SECRET and CARDIA have recently suggested that skeletal muscle fat could play a direct role in increasing cardiometabolic risk (319–322). However, while ectopic fat in skeletal muscles is often associated with metabolic disease, highly trained athletes have been reported to have comparable amounts of skeletal muscle fat as subjects with T2DM, yet their tissue remains highly insulin sensitive (323). This phenomenon has been called “the athlete's paradox” and is likely due to the high energy demands of skeletal muscle in extremely fit athletes.

##### Ectopic fat in the heart

Obesity and T2DM are both independently associated with fat accumulation in the heart (324), rendering ectopic fat in the heart as a strong predictor of CVD (325, 326), particularly in subjects with T2DM (327). Similar to the liver, excess circulating FFA can also lead to increased triglyceride deposition in the heart. Cardiac tissue mainly utilizes FFA for metabolism, but when delivered in excess of basal myocardial fatty acid oxidation rates can also lead to the accumulation of lipotoxic products (328). In addition to ectopic cardiac myocyte lipid storage, excess FFA can be stored in epiWAT, pericardial fat (between the visceral and parietal pericardia), or PVAT (329). PVAT in particular has a major impact on vascular homeostasis. As a source of several vasoactive mediators, PVAT influences vascular contractility. Healthy PVAT is thought to be a largely anti-inflammatory tissue (330), with characteristics akin to BAT in the areas surrounding the thoracic aorta in particular (331). However, in the setting of obesity, dysfunctional PVAT releases predominantly vasoconstrictive and proinflammatory mediators that negatively influence vascular homeostasis (332–334). Similarly, epiWAT is a source of bioactive molecules that negatively impact cardiac rhythm and perpetuate an atherogenic environment in obesity (335). Patients with T2DM express higher levels of the LDL and very low-density lipoprotein (VLDL) receptors in epiWAT than non-diabetic control subjects (336), suggesting that altered lipid metabolism in epiWAT could be associated with T2DM.

##### Ectopic fat in the pancreas

Mounting evidence suggests that excessive fat in the pancreas is associated with an increased risk of metabolic disorders, with reports that nearly 2/3 of the obese population has excessive pancreatic fat (337). Recent studies have connected ectopic pancreatic fat with β-cell dysfunction and T2DM (338–340), which in turn is associated with an increased risk of CVD. Therefore, lipotoxic lipid intermediates may also play a role in increasing the risk of CVD by elevating levels of pancreatic fat, thus leading to T2DM (341). In contrast to skeletal muscle, ectopic pancreatic fat is characterized mostly by adipocyte infiltration rather than intracellular lipid accumulation (342). The accumulation of fat in the pancreas also has been reported to accelerate acute pancreatitis due to increased levels of lipolysis and inflammation (343, 344).

### Brown and Beige Adipose Tissue, Inflammation, and the Metabolic Syndrome

Compared with healthy lean controls, obese subjects display reduced BAT content, identified as tissue that actively takes up 2-[^18^F]fluoro-2-deoxyglucose (FDG) (345). This reduction in active BAT mass appears to be more prevalent in visceral obesity (346, 347). Concurrently, individuals with detectable BAT activity display lower blood glucose, triglyceride and FFA levels, lower glycated hemoglobin (Hb1Ac) levels, and higher HDL cholesterol levels than people with no detectable BAT (348, 349). As discussed in other sections, BAT acts as an important “sink” for excess blood glucose and FFA disposal. Thus, loss of BAT function in association with obesity could contribute to the development of insulin resistance and hyperlipidemia. It has been shown that while cold exposure can activate BAT to a certain degree in obese subjects and those with T2DM, the levels of BAT activation achieved are substantially lower than in healthy lean subjects (350, 351). While BAT is largely resistant to the development of mild obesity-induced local inflammation, BAT inflammation becomes quite pronounced with stronger obesogenic insults (352). Such inflammation can directly upset the thermogenic potential of BAT by impairing its ability to take up glucose (described in more detail in later sections) (353, 354). Whether individuals who inherently possess less active BAT are more prone to obesity and facets of the metabolic syndrome or whether these pathological conditions themselves reduce BAT activity requires further investigation. Regardless, it is still widely believed that strategies that augment BAT or beige activity could represent viable therapeutics to combat metabolic syndrome (355, 356). Efforts to enhance BAT activation in humans consist of intermittent regular cold exposure, introduction of β_3_-adrenergic receptor agonists, and exercise (29, 357). However, robust reductions in body weight in humans have not yet been shown to be clinically significant when BAT is activated (358), necessitating further mechanistic studies to elucidate whether BAT activation is a viable target for metabolic improvement in humans.

Whether BAT undergoes similar immune cell changes as WAT under obesogenic conditions is still not clear. In one study, BAT isolated from mice made obese by 13 weeks of high fat diet feeding displayed lower mRNA expression of inflammatory genes, lower immunostaining for macrophage markers F4/80 and CD68, and lower macrophage content by FACS analysis (331). However, subsequent studies have shown that BAT becomes inflamed in obese mice, with increased mRNA expression levels of inflammatory markers *Tnf* and *Emr1* (the gene that encodes the macrophage marker F4/80) (359–361). Such BAT inflammation reportedly lowers the thermogenic potential of this tissue (359), presumably due to increased local insulin resistance (360, 362), which could reduce the glucose and fatty acid oxidizing capacity of BAT.

Similar to BAT, beige adipocyte quantity and functionality appear to be sensitive to local inflammation. A study in which IkB kinase (IKK, an enzyme that is required for NFκB activation and subsequent inflammatory cytokine transcription) was inactivated in mice, not only blunted adipose tissue inflammation and body weight gain, but enhanced WAT browning (363). Similarly, inhibiting a major intracellular mediator of toll-like receptor 4 (TLR4) signaling, interferon regulatory factor 3 (IRF3), blunted WAT inflammation and augmented WAT browning (364). Moreover, it has been shown that the immune cell infiltration of subcutaneous WAT that accompanies obesity directly interferes with the differentiation and/or recruitment of beige adipocytes (365). Thus, accumulating evidence suggests that obesity-associated inflammation hinders the thermogenic and insulin sensitizing effects of both BAT and beige adipocytes.

## Obesity and Insulin Resistance

Abundant evidence indicates that adiposity and adipose tissue inflammation are associated with insulin resistance, which refers to a reduced response to binding of insulin to its receptor in peripheral tissues such as adipose tissue and skeletal muscle. This differs from glucose effectiveness, which is uptake of glucose by peripheral tissues in an insulin-independent manner. Insulin inhibits hepatic glucose output and stimulates lipogenesis in the liver, both of which are reduced in the presence of insulin resistance. Such desensitization of insulin signaling pathways also inhibits glucose uptake in peripheral tissues and stimulates lipolysis in adipose tissue. To compensate for reduced insulin sensitivity, insulin secretion is increased in order to maintain euglycemia. If the pancreatic beta cells are unable to secrete sufficient insulin to compensate for the reduced insulin sensitivity (termed beta cell dysfunction), hyperglycemia will ensue, leading to glucose intolerance and eventually T2DM (366). While the precise mechanisms that lead to beta cell dysfunction are not completely understood, ectopic fat accumulation may contribute, as discussed earlier. Nonetheless, ample evidence suggests that excess adiposity and adipose tissue inflammation contribute to insulin resistance [reviewed in (64, 367)]. Many studies have demonstrated that excess adiposity is correlated with insulin resistance in humans. Cross-sectional studies in men of European, Asian Indian, and American descent have shown that total, visceral, and subcutaneous adiposity, BMI, and waist circumference are all negatively associated with insulin sensitivity (368, 369). As noted earlier, adiposity, especially visceral adiposity, is characterized by adipose tissue inflammation.

Several hypotheses have been put forth to account for the relationship between adipose tissue inflammation and insulin resistance. These include production of pro-inflammatory cytokines by adipocytes and adipose tissue macrophages (discussed previously in the section on WAT Inflammation), excess FFA, decreased adiponectin, increased resistin and retinol binding protein, ceramide accumulation, and ectopic fat accumulation in liver and skeletal muscle (367).

### Free Fatty Acids

It was initially hypothesized that excess adiposity promoted insulin resistance due to the accelerated release of FFA by obese adipocytes, which inhibit insulin signaling in liver and muscle due to excessive lipotoxicity and/or ectopic fat storage in these tissues (64), and also contribute directly to beta cell dysfunction (366). It has been shown that adipose tissue mass correlates with circulating FFA in obese humans, with a tendency for individuals with visceral adiposity to have higher FFA turnover (370–372). It has also been reported that individuals with T2DM tend to have elevated FFA levels over non-diabetic controls (373), an effect found to correlate more strongly with insulin sensitivity rather than obesity (374). Consistent with this, one study reported that FFA levels were lower in MHO subjects than those with MUHO (375). In addition to dysregulated energy metabolism, disruption of the endocrine function of obese adipose tissue has now been shown to contribute to insulin resistance, described in more detail below.

### Adipokines

Adipocytes in obesity simultaneously secrete lower levels of adiponectin and elevated levels of cytokines and chemokines, such as TNFα, IL-6, MCP-1, and SAA. Not only is there evidence that such inflammatory cytokines contribute directly to insulin resistance in hepatocytes and myocytes (366), they also directly inhibit adiponectin production from adipocytes (376).

There is evidence that hypoadiponectinemia plays a role in obesity-associated T2DM (377–380). Subjects with T2DM exhibit reduced circulating adiponectin levels (379, 380); similarly, MHO subjects have higher circulating adiponectin than those with MUHO (206). Obese mice that are deficient in leptin (*Lep*^*Ob*/*Ob*^ mice) that are engineered to overexpress adiponectin are protected from obesity-associated insulin resistance, despite having elevated adiposity (97). This may be explained by the nature of adipose tissue expansion in these transgenic mice, which had smaller, less inflamed adipocytes and less liver fat content. Similarly, administration of recombinant adiponectin improved glucose tolerance and insulin sensitivity in obese high fat diet-fed or *Lepr*^*db*/*db*^ mice (377).

As discussed in earlier sections, FGF21 is a hormone produced by the liver as well as adipocytes that exerts insulin-sensitizing effects. However, recent evidence has paradoxically suggested an association between serum FGF21 levels and obesity-associated metabolic syndrome (145, 381). FGF21 levels have been reported to be 2-fold higher in MUHO when compared to MHO (148). Moreover, subjects with T2DM were reported to have significantly higher plasma levels of FGF21 than insulin-sensitive controls, with FGF21 levels positively correlated with BMI, HOMA-IR, and Matsuda index, suggesting a strong correlation with insulin resistance (157). Plasma FGF21 levels also correlated strongly with visceral, epicardial, hepatic, and skeletal muscle ectopic fat levels, measured using 64-slice multidetector CT scanning (157). Given that FGF21 has been shown to improve insulin sensitivity and promote negative energy balance (382, 383), some have suggested that obesity and associated metabolic syndrome represent an “FGF21-resistant” state (146). This conclusion was reached based on some observations that circulating FGF21 levels are increased in obesity, with lower FGF21 receptor expression levels on target tissues such as adipose tissue (146, 384). However, this notion has been challenged by evidence that obese subjects are equally responsive to pharmacological administration of FGF21 (384, 385). Thus, it has now been proposed that obesity-associated FGF21 is increased as a compensatory mechanism to preserve insulin sensitivity (386). As such, a clear role for adipocyte-derived FGF21 in obesity and associated metabolic syndrome is still lacking.

### Adipose Tissue Plasticity

Evidence suggests that ineffective adipose expansion promotes local inflammation and an insulin resistant phenotype (387). However, sufficient adipogenesis and hyperplasia (i.e., the ability to distribute fat among newly differentiated adipocytes without the need for significant hypertrophy) mitigates such inflammation and subsequent insulin resistance (388). Thus, strategies to increase the recruitment of adipocyte progenitor cells to expand adipose tissue by increasing adipose cell numbers could be protective against the metabolic consequences of obesity.

A key structural and functional component of adipose tissue is made up of extracellular matrix (ECM) molecules, including collagen and proteoglycans such as versican and biglycan, among others (389). Adipose tissue makes large quantities of ECM during active remodeling, as would occur during WAT expansion in obesity (390–392). Obese animal models and humans with obesity and/or T2DM exhibit large increases in visceral WAT ECM content, which can contribute to the local inflammatory milieu (393, 394). To date, most studies of WAT ECM function have centered around collagen, which can form a scaffold that constrains adipocyte expansion due to mechanical stress (391, 392, 395). Targeting ECM components to release adipocytes from such constraints due to excessive ECM production could potentially alleviate the ectopic accumulation of fat that drives the metabolic syndrome.

### Visceral Adipose Tissue vs. Hepatic Lipid Accumulation?

While the majority of adipose tissue in humans is localized subcutaneously (396), the volume of visceral adipose tissue is believed to be a strong predictor of insulin resistance (397), independent from subcutaneous fat quantity (397, 398). The association between insulin resistance and visceral adipose mass is particularly striking in certain ethnic populations, with T2DM rates of 46.6% in Filipino, 14.7% rates in African American, and 9.8% rates in Caucasian populations (398), suggesting a strong genetic component. While visceral adiposity is positively associated with insulin resistance, there is evidence to suggest that it may not be a causal factor. Other conditions associated with visceral adiposity, such as hepatic fat content, may instead drive insulin resistance (292, 399). Some clinical studies have dissociated the glucose metabolic effects of visceral adiposity from hepatic lipid accumulation. In one such study, significant differences in insulin sensitivity in the liver, skeletal muscle, and adipose tissue were reported in obese human subjects who differed in hepatic lipid content, with no such differences observed in obese subjects who differed in visceral adiposity (291). Similarly, in a study in which obese subjects were matched for liver fat content, no differences in indices of glucose metabolism were noted (293). Insulin-sensitive MHO individuals tend to have lower visceral and intrahepatic fat accumulation than their MUHO counterparts (203, 400, 401), providing further evidence that these fat depots contribute to insulin resistance. Collectively, while visceral adiposity and hepatic fat content are both strongly associated with whole-body and tissue-specific insulin resistance, hepatic lipid accumulation may play a more direct role in negatively modulating glucose homeostasis.

### Subcutaneous Adipose Tissue

Many studies have suggested that fat distribution is strongly associated with insulin resistance, with visceral adiposity being the strongest predictor of insulin resistance (198, 402, 403). While the detrimental effects of visceral and hepatic lipid accumulation on glucose metabolism are clear, it is also becoming increasingly appreciated that lower body subcutaneous adiposity may be metabolically protective (404–406). Large-volume liposuction of subcutaneous WAT has shown little to no metabolic benefit in human trials (407). Gluteofemoral adipose mass is positively associated with insulin sensitivity in humans, coupled with a slower rate of lipolysis and subsequent FFA release, lower levels of inflammatory cells and cytokines, and elevated adipokines such as leptin and adiponectin (404). Evidence from animal models has suggested that transplantation of subcutaneous WAT into the visceral cavity of recipient mice promotes less body weight and adiposity gain than transplantation with visceral WAT, resulting in greater insulin sensitivity in the liver and endogenous WAT (408). Taken together, a growing body of evidence suggests that adipose tissue and ectopic lipid distribution contribute to whole-body glucose homeostasis.

### Brown and Beige Adipose Tissue

With the purported potential to improve glucose homeostasis, interest in BAT and beige adipose tissue as therapeutic targets has increased in recent years. Studies in rodents in which BAT is transplanted into diseased mouse models have shown that transplanted BAT improves insulin sensitivity, glucose metabolism, and obesity (409–411), likely mediated by batokine effects. While the predominant energy source that contributes to brown adipocyte heat production derives from fatty acids (412) (~90%), with only ~10% of energy derived from glucose, BAT is still regarded as having a strong impact on glucose homeostasis. As a highly metabolically active organ, BAT contributes to glucose clearance by taking up relatively large amounts of glucose from the circulation, thus reducing insulin secretion by pancreatic β-cells (413). Indeed, individuals that possess detectable BAT have lower fasting glucose concentrations than those without active BAT (414). Glucose disposal through activated BAT occurs by both insulin-dependent and insulin-independent mechanisms (415). For example, the cold exposure-mediated influx of glucose into active BAT has been suggested to be an insulin-independent process (416–418). However, as the insulin receptor is highly expressed in BAT tissue, it is considered to be one of the most sensitive insulin target tissues and thus an important organ for glucose disposal (413). BAT activation further enhances insulin signaling in BAT itself by augmenting insulin-independent glucose uptake associated with thermogenesis and glucose uptake due to insulin signaling. Thus, strategies that activate BAT and beige adipose tissue have the capacity to improve insulin resistance by clearing excess glucose (419–421).

## Links Between Obesity, Insulin Resistance, and CVD

### Obesity as a Risk Factor for CVD

**Figure 2:**
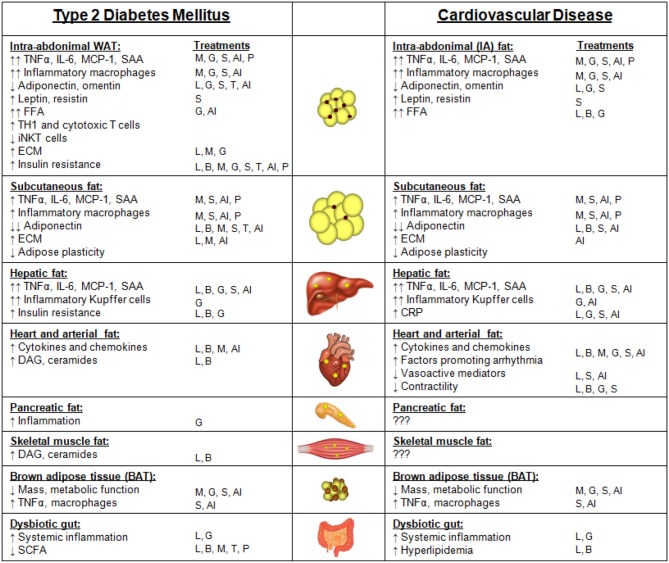
Adipose depots and ectopic fat sites and their features that contribute to type 2 diabetes mellitus (T2DM) or cardiovascular disease (CVD). Features of intra-abdominal white adipose tissue (WAT), subcutaneous fat, hepatic fat, heart and arterial fat (inclusive of epicardial, pericardial, and perivascular fat), pancreatic fat, skeletal muscle fat, brown adipose tissue, and a dysbiotic gut that contribute to either T2DM or CVD. Arrows indicate changes in comparison with subjects without T2DM or CVD. The T2DM treatment strategies that have been reported to improve each adipose depot feature are listed under “treatments.” Treatments: weight loss due to lifestyle changes (L); weight loss due to bariatric surgery (B); metformin (M); GLP-1 receptor agonists (G); SGLT-2 inhibitors (S); thiazolidinediones (TZDs, T); anti-inflammatory approaches (AI); microbiome modulation with pre- or pro-biotics (P).

Several pathologic conditions, including hypercholesterolemia and systemic inflammation, are hypothesized to drive atherosclerotic CVD. With a primary function of sequestering lipotoxic lipids and the known potential for chronic inflammation, obese adipose tissue has emerged as a potential player in the regulation of these atherogenic factors. Obesity has been officially classified as an independent risk factor for CVD by the American Heart Association since 1995, meaning that obesity treatment is likely to lower the incidence of CVD (422). As alluded to in previous sections, people with MHO are at a lower risk of experiencing cardiovascular events than people with MUHO (423), yet those without obesity are at a considerably lower risk for future events. Thus, even a moderate level of weight loss, if sustainable, could potentially lower the risk of adverse CVD events (120). However, some studies have shown that individuals with established CVD and heart failure with moderate degrees of obesity present a more favorable prognosis than those who are normal or underweight, a situation that has been termed the “obesity paradox” (424, 425). Possible reasons include confounding factors such as smoking and the presence of co-morbidities that are associated with lower body weights, or the use of BMI rather than measures of visceral obesity for most studies on the obesity paradox. Despite the obesity paradox in those with established CVD, the following sections will provide information regarding potential links between obesity T2DM and CVD. The various features of adipose tissue depots, including ectopic fat, and how they contribute to T2DM and CVD are summarized in Figure 2. Notably, there are many similarities between adipose depot characteristics that contribute to both T2DM and CVD.

### Adipose Depot-Specific Links With CVD

#### Visceral and Subcutaneous White Adipose Tissue

The accumulation of visceral fat in obesity is associated with the metabolic syndrome, its associated CVD risk factors, and an increased risk for clinical CVD (426). This distribution of WAT has been shown to have the greatest effect on CVD risk and mortality among patients with normal body weight (427). The risk of CVD in the metabolic syndrome has been considered to result from the presence of multiple CVD risk factors such as dyslipidemia (hypertriglyceridemia, an excess of small, dense LDL particles and reduced HDL-cholesterol levels), hypertension, dysglycemia, and a thrombogenic profile that have been reviewed elsewhere (428–430). However, there are several additional potential mechanisms by which visceral WAT might contribute directly to CVD that involve FFA, insulin resistance, and inflammation. Visceral WAT has higher lipolytic activity than subcutaneous WAT due to its having fewer insulin receptors, and thus is a significant source of FFA. Visceral-derived FFA can directly impact the liver via the portal vein, facilitating FFA uptake by the liver and subsequent hepatic insulin resistance. Similarly, excess FFA from visceral fat might directly impair lipid metabolism and lead to dyslipidemia, which increases CVD risk. In obese diabetic subjects, plasma FFA levels have been shown to be elevated compared to BMI-matched non-diabetic subjects (373), supporting the notion that insulin resistance further elevates circulating FFA levels. Moreover, the incidence of T2DM is nearly doubled in patients with the highest levels of FFA (90th percentile) when compared with subjects with the lowest FFA levels (10th percentile) (431). In one study, obese T2DM subjects who had undergone overnight fasting during pharmacological inhibition of lipolysis exhibited improved insulin sensitivity and glucose tolerance (432), providing further evidence for an inhibitory effect of FFA on insulin sensitivity.

The adipokine profile of visceral WAT also contributes substantially to its association with CVD risk. Obese visceral WAT primarily secretes inflammatory cytokines such as resistin, TNFα, IL-6, IL-1β, MCP-1, and SAA, with reduced levels of adiponectin (433). Plasma adiponectin levels are decreased in patients with CVD (434). Adiponectin is believed to contribute to CVD protection by several mechanisms, including the reduction of lipid levels, repressing expression of inflammatory mediators such as VCAM, ICAM, E-selectin, TNFα, and IL-6, and by acting directly on the heart to improve ischemic injury by activating AMPK and subsequently increasing energy supply to the heart (435–438). Adiponectin also stimulates endothelial nitric oxide synthase (eNOS), which maintains healthy vascular tone (439, 440). Thereby, adiponectin would play a protective role in the development of CVD. Conversely, leptin levels are positively associated with acute myocardial infarction, stroke, coronary heart disease, chronic heart failure, and left cardiac hypertrophy (441–445), although the reasons for this remain largely unknown. Leptin receptors are expressed in the heart, indicative of an important impact of direct leptin signaling (446). Resistin is positively associated with systemic inflammatory markers (447), upregulates endothelial expression levels of VCAM-1 and endothelin-1 (448) and promotes the proliferation of smooth muscle cells (449). Resistin also associates positively with coronary artery calcification levels, and negatively with HDL cholesterol (450). Thus, adipose-derived resistin levels could be used to predict the severity of coronary atherosclerosis (450). Similarly, cytokines and chemokines such as those secreted from obese visceral WAT can induce expression of endothelial adhesion molecules (451), recruit macrophages (452), increase thrombosis (453), and reduce vasoreactivity (454), and are positively associated with cardiovascular events (249, 455). While visceral WAT-derived cytokines are associated with these CVD-inducing processes, it is important to note that the direct contribution from visceral WAT is not currently known, as these are also secreted from other tissues.

As discussed in previous sections, in addition to cytokines and exclusive adipokines, WAT is also a source of FGF21. While the liver is considered to be the major source, adipocytes have also been shown to produce FGF21 to varying degrees in response to various stimuli. In addition to its associations with obesity and T2DM, FGF21 levels have also been associated with increased risk for CVD (456–460). Subjects with CVD that also had diabetes exhibited even higher levels of FGF21 (459), suggesting an important role in diabetes-accelerated atherosclerosis. In particular, FGF21 levels have been shown to positively correlate with hypertension and triglyceride levels, and to negatively correlate with HDL-cholesterol levels (461). One study by Lee et al. suggested that plasma FGF21 levels are associated pericardial fat accumulation (462), which suggests that ectopic fat could be a source of FGF21 in metabolic disease. Further studies are needed to discern whether adipocyte- or hepatic-derived FGF21 contribute to these effects. In stark contrast to these effects of physiological FGF21, pharmacological administration of FGF21 in humans and non-human primates reduces blood glucose, insulin, triglycerides, and LDL cholesterol, and increases HDL cholesterol (142, 463, 464). Thus, there is a disconnect between the physiological and pharmacological effects of FGF21 that requires further study.

#### Epicardial, Perivascular, and Brown Adipose Tissues

It is becoming increasingly clear that adipose tissue expansion contributes directly to obesity-associated cardiovascular disease risk (465). Obesity is accompanied by not only excess visceral adiposity, but also by excess epicardial and perivascular WAT (466). Due to their proximity to the heart, coronary arteries, and other major arterial blood vessels that are prone to atherosclerosis, it is not surprising that epiWAT and PVAT are important regulators of cardiac and vascular. The respective sizes of these adipose depots are associated with risk factors for the metabolic syndrome, including elevated visceral fat content, blood glucose, hypertension, systemic inflammation, insulin resistance, circulating LDL levels, mean arterial pressure, and atherosclerosis (19, 467–471), as well as adverse cardiovascular events (472–475). The mechanisms behind these associations include increased secretion of pro-inflammatory cytokines, vasoactive factors, and vascular growth factors (476–478); increased release of lipotoxic FFA (479, 480); increased macrophage content (481); increased oxidative stress (482); and decreased secretion of adiponectin (483), which are triggered by obesity. In a prospective cohort of patients with aortic stenosis, a positive association between epiWAT volume and left ventricular mass was found (484), suggesting that in addition to changes in adipokine secretion, epiWAT could negatively influence cardiac function by placing a restrictive burden on the heart. Mechanisms by which PVAT influences CVD are more nuanced and complex. As an adipose depot that features some characteristics of both WAT and BAT, and with different functions depending on the anatomical location (i.e., thoracic vs. abdominal aortic PVAT), PVAT can play either a cardioprotective or a pathological role (24). As obesity progresses, PVAT can become dysfunctional in that it more resembles WAT, and contributes to a pro-inflammatory and lipotoxic microenvironment that promotes atherosclerosis (485). Similarly to healthy PVAT, BAT provides atheroprotection by serving as a protective “buffer” for the vasculature against lipotoxic FFA (486). However, BAT can become dysfunctional as obesity progresses, undergoing a phenotypic “whitening” switch that promotes atherosclerosis (487). Thus, while PVAT and BAT play atheroprotective roles in healthy individuals, obesity promotes dysfunction of these depots, blunting this protective effect against CVD.

## Strategies for Reducing T2DM and/or CVD Risk That Impact Adipose Tissue

Strategies for weight loss are multi-faceted, including combinations of diet and lifestyle modifications, pharmaceutical therapy, and various forms of bariatric surgery (488). While there is some debate over this, it is generally believed that small degrees of weight loss in MUHO obese populations can have a dramatic impact on cardiometabolic health (489, 490); thus, strategies that improve obesity are likely to also decrease risk factors for CVD. Similarly, CVD treatment strategies are centered around a combination of pharmaceutical use and lifestyle modifications, which also impact adipose tissue. In this section, we will describe the effects that various CVD treatment strategies have on adipose tissue metabolism and inflammation. How these treatment strategies impact the contributions of particular adipose depot features to T2DM and CVD are listed in Figure 2.

### Weight Loss

#### Lifestyle Modifications: Dietary Changes

As most patients with T2DM and/or CVD are overweight or obese, weight loss often is the first strategy to reduce the severity of T2DM and/or CVD. Traditional methods prescribed for weight loss include restricting food intake and increasing energy expenditure. Despite a large number of fad diets that dictate particular proportions of dietary fat, protein, and carbohydrates to facilitate weight loss [summarized in (488, 489)], the simple fact remains that for weight loss to occur, energy balance *must* be negative. Thus, energy intake must be less than energy expended, which includes resting energy expenditure, physical activity, and the thermic effect of food.

It has been previously reported that for every kilogram of body weight lost due to dietary restrictions, visceral adiposity is reduced by around 2–3% (491). Subsequently, additional studies have shown that modest weight loss due to dietary changes in people with overweight or obesity is due to roughly equivalent fat lost from subcutaneous and visceral depots, while the addition of exercise leads to more weight loss from subcutaneous fat as well as loss of ectopic skeletal muscle fat (492–495). The loss of visceral fat is associated with reduced CVD risk factors, including reduced systemic inflammation, total cholesterol, LDL cholesterol, and triglycerides (493, 496), as well as reduced fasting glucose and insulin levels (496, 497). A *post-hoc* analysis from the Look AHEAD study showed that weight loss of ~10% in overweight or obese subjects with T2DM yielded a 21% lower risk of the primary outcome (including CVD-related death, non-fatal acute myocardial infarction, non-fatal stroke, or hospital admission for angina) (498). As the subjects recruited for the Look AHEAD trial had T2DM, this and other *post-hoc* analyses suggest that weight loss in T2DM subjects also lowers the risk of CVD events (499, 500).

#### Lifestyle Modifications: Including Exercise

It is well established that aerobic exercise increases fuel mobilization from adipose tissue by increasing lipolysis and subsequent FFA mobilization, which ultimately decreases adiposity and adipocyte size (501–504). Such enhanced fuel mobilization is thought to be highest for visceral WAT (505). Several studies have shown that a high level of fitness (defined by a high activity level with maximal oxygen uptake) negatively associates with visceral adiposity (506–508), even in subjects with obesity and/or T2DM, suggesting that aerobic exercise contributes to a favorable adipose distribution profile that reduces the risk of metabolic syndrome. Hepatic fat is also mobilized and decreased following intense aerobic exercise (509). Studies in mice suggest that not only visceral fat mass is lost with regular exercise, but subcutaneous and brown fat mass are also diminished (510). As expected with fat loss, exercise is coincident with reduced plasma and adipose tissue leptin levels (511–516). The effects of exercise-induced fat loss on adiponectin levels are less clear, with some studies showing no changes in circulating adiponectin levels (517–519), some showing increased plasma adiponectin (520–522), and others showing increased subcutaneous WAT expression of adiponectin mRNA (523–525). A meta-analysis showed that pediatric subjects with obesity exhibit reduced resistin levels following aerobic exercise (526). Little is known about the impact of exercise on FGF21 in obese humans, but one study suggested that aerobic exercise training in obese women reduced circulating FGF21 levels (527). By contrast, studies in rodents have shown that circulating FGF21 levels are not altered by exercise in obese animals (528). Collectively, such exercise-induced changes to WAT distribution and adipokine secretion likely facilitate the observed improvements in insulin sensitivity and CVD risk factors observed with exercise.

While many studies have reported that exercise training increases subcutaneous WAT browning in rodent models of obesity (529–532), there is limited data to support this in humans. Many studies have shown that there is no effect of aerobic exercise training to recruit beige adipocytes in humans (533). However, one study compared subcutaneous WAT from lean, sedentary young men with age- and weight-matched endurance-trained men and reported no differences in beige markers such as *UCP1, PGC1A*, or *CIDEA* (534). Another study found evidence of subcutaneous WAT browning (i.e., increased *UCP1* and *CPT1B* expression) in overweight sedentary individuals that had undertaken a 12-week bicycle training program (535). There is some debate about what role brown or beige adipose tissue would play in exercise, if it indeed occurs. It is known that BAT and beige activity is increased when thermogenesis is required, and exercise is a highly thermogenic activity that raises core body temperature, so it is not immediately clear why exercise would increase BAT and/or beige activity. Exercise is known to activate the sympathetic nervous system, which also activates BAT to quickly release stored energy, so it is possible that BAT activation is secondary to exercise-induced sympathetic activation (536). Nevertheless, further studies are needed to determine what role if any BAT and/or beige adipocytes play in mediating the metabolically beneficial effects of exercise.

Loss of adipose tissue mediated by dietary changes, exercise, liposuction, or bariatric surgery (discussed in the section on Bariatric Surgery) is accompanied by decreased markers of adipose tissue and systemic inflammation (537, 538). Weight loss achieved through calorie restriction and/or exercise resulted in decreased systemic IL-6, CRP, TNFα, MCP-1, soluble intercellular adhesion molecule-1 (ICAM-1), and vascular cell adhesion molecule-1 (VCAM-1) (273, 539–542). Fat loss by liposuction yielded similar changes in systemic inflammatory markers in one study (543), but did not improve plasma cytokine levels in another (407). The removal of visceral fat from Zucker diabetic fatty rats resulted in dramatic reductions in systemic cytokines (544); this suggests that removing visceral fat, rather than the subcutaneous fat that is routinely removed during liposuction, is more advantageous in terms of resolving inflammation. Many studies also have shown that weight loss following bariatric surgery leads to reductions in systemic inflammatory markers (545), with notable reductions in adipose tissue inflammatory cytokine and macrophage expression (546–548). However, some similar studies do not show improvements in adipose tissue inflammation following various weight loss modalities, such as bariatric surgery or very low-calorie diets (549–551). It has been suggested that pronounced weight loss over time can lead to improvements in adipose tissue inflammation that were not observed in the same subjects following acute moderate weight loss (552). This implies that adipose tissue inflammation during the initial stages of weight loss could be required for the pronounced adipose tissue remodeling required for fat loss (284, 553).

#### Medications Indicated for the Treatment of T2DM That Lead to Weight Loss

##### Metformin

Metformin is the most commonly prescribed medication to treat T2DM, particularly in subjects with obesity (554). Metformin has been proposed to lower blood glucose levels through suppression of gluconeogenesis in the liver, activation of AMP-activated protein kinase (AMPK), inhibition of the mitochondrial respiratory chain (complex 1), and by unknown mechanisms in the gut (555, 556). Thus, the precise mechanisms by which metformin lower blood glucose are complex and still evolving. While some diabetes medications have adverse effects on body weight, patients taking metformin often lose a small amount of weight [reviewed in (557)]. Studies in T2DM suggest that metformin may reduce body fat stores and promote a more metabolically healthy fat distribution (558–560). The effect of metformin on adiposity may be partially due to reported nausea and anorexic effects of the drug (561–563). Metformin has been shown to decrease visceral WAT mass, potentially by promoting fatty acid oxidation and/or adaptive thermogenesis (564). With much recent attention focused on BAT as a potential target for obesity treatment, it has recently been shown that BAT is an important effector organ in the glucose-lowering effects of metformin (565). Some studies have reported increases in omentin following metformin therapy, which could be due to visceral fat loss (566). Metformin also reduces hepatic steatosis through inhibition of ApoA5 and steroyl-CoA desaturase-1 (SCD1) which combine to limit *de novo* lipid synthesis, which is partially mediated by its actions on AMPK and liver X receptor (LXR) activity (567, 568). It also has been suggested that metformin reduces ECM remodeling that is dysregulated in obesity (see previous section on adipose tissue plasticity), and reduces lipogenesis (564).

In addition to the increasingly recognized anti-obesity effects of metformin, its ability to improve CVD risk is also becoming apparent (569). A recent meta-analysis suggested that metformin could contribute to a 16% decrease in all-cause mortality, but may also contribute to a 48% increased risk of stroke (570). The mechanism may include improvements in the lipid profile, such as mild reductions in plasma VLDL cholesterol and triglycerides with slight elevations in HDL cholesterol (571). In addition, metformin has been shown to have anti-inflammatory properties, reported to reduce circulating CRP and MCP-1, reduce NFκB activity, and to reduce advanced glycation end products (AGE) (572–576).

##### GLP-1 receptor agonists

Glucagon-like peptide-1 (GLP-1) is a peptide hormone that is continuously secreted at low levels during fasting by intestinal L cells. Consumption of a meal enhances GLP-1 secretion, which functions to reduce plasma glucose levels by stimulating insulin secretion from pancreatic beta cells. In addition, GLP-1 receptors are abundant in brain areas that control food intake regulation, such as the hypothalamus, where GLP-1 functions to reduce the drive to eat (577, 578). Thus, several GLP-1 receptor agonists have been developed to mimic the glucose-lowering and anorexic effects of GLP-1 to treat obesity and T2DM.

Liraglutide, a GLP-1 receptor agonist, has shown efficacy in not only glucose control, but also in promoting weight loss and reduced waist circumference based on results from the Liraglutide Effect and Action in Diabetes (LEAD) study (579–581). Liraglutide has also been shown to reduce total adiposity, and specifically visceral fat mass (582, 583). While initially described as being devoid of GLP-1 receptors (584), it has now been confirmed that adipocytes express the GLP-1 receptor (585, 586). Adipose tissue may therefore be an additional target for GLP-1 receptor agonists to promote adipose remodeling by unknown mechanisms. In addition to its effects on body weight and glucose metabolism, GLP-1 receptor agonists may also provide protection against CVD (587). The Liraglutide Effect and Action in Diabetes: Evaluation of Cardiovascular Outcome Results (LEADER) trial showed that liraglutide lowered the risk of myocardial infarction and non-fatal stroke among patients with T2DM that had high CVD risk (587). GLP-1 receptor agonist treatment has been shown to protect against atherosclerosis in animal models and in humans, potentially by lowering plasma lipids and by reducing circulating CRP and soluble ICAM-1 levels (588–590). Liraglutide, when administered in combination with metformin as indicated for the treatment of T2DM, has been shown to reduce epicardial WAT volume with simultaneous increased omentin expression (591). Thus, liraglutide may provide cardioprotection through reduced levels of ectopic fat, lipids, and inflammation.

##### SGLT-2 inhibitors

Inhibitors of the sodium-glucose cotransporter 2 (SGLT-2) have been shown to reduce blood glucose levels in subjects with T2DM by enhancing urinary glucose excretion (592). The SGLT-2 inhibitor empagliflozin, alone and in combination with the GLP-1 receptor agonist liraglutide, has been shown to reduce CVD risk (593), as well as cardiovascular death to a greater extent than statins alone (488). Empagliflozin also is associated with decreased hypertension, reduced arterial stiffness, and decreased vascular resistance (594, 595). In both rodents and humans with non-alcoholic fatty liver disease, SGLT-2 inhibitors have been shown to reduce ectopic liver fat by blunting *de novo* hepatic lipogenesis (596–599), with reduced alanine transaminase (ALT) and aspartate transaminase (AST) levels (600), two markers of hepatic metabolic stress. Furthermore, empagliflozin is associated with weight loss in humans when administered in combination with other therapeutics, such as metformin, thiazolidinediones, and sulfonylureas (601–603). In rodents, SGLT-2 inhibitors have been shown to suppress high fat diet-induced weight gain and to markedly reduce obesity-induced inflammation in WAT, potentially by increasing fat oxidation and the recruitment of beige adipose tissue (604, 605). Thus, in addition to correcting hyperglycemia, SGLT-2 inhibitors can also impact adipose tissue physiology; whether this is through direct or indirect mechanisms remains to be elucidated.

##### Bariatric surgery

Bariatric surgical techniques, including Roux-en-Y gastric bypass (RYGB) and sleeve gastrectomy, are widely acknowledged to be the most effective treatment strategies for obesity, achieving relatively low levels of obesity remission (606). Within the first year of surgery, some patients experience the loss of around half of their adipose tissue mass (607), often with roughly equivalent losses from subcutaneous and visceral WAT (608, 609). As weight loss progresses, studies have shown that later weight loss is largely from visceral depots (610–612), an effect that correlates with the degree of diabetes remission (609). It has also been reported that ectopic skeletal muscle and pancreatic fat are reduced following bariatric surgery (610, 613, 614), which could contribute to improved glucose metabolism. Studies in humans have reported that subcutaneous adipocytes become smaller following bariatric surgery, resembling adipocytes from lean individuals, but that total adipocyte number remains unchanged (615, 616). Little is known regarding the size and number of visceral adipocytes, which are extremely difficult to sample from humans. As expected with reduced adipocyte size, leptin levels have been shown to decrease following bariatric surgery, while adiponectin has been shown to increase in some studies (617, 618), but not in others (549, 550). Whether changes in adipokine secretion are important for the sustained metabolic improvements following bariatric surgery or whether they simply reflect the adipose remodeling remain to be elucidated. However, it is worth noting that one study has shown that adiponectin levels are elevated only 2 weeks following bariatric surgery, before significant weight loss has occurred, suggesting that adipokine responses may be independent from weight loss (619).

Following bariatric surgery, obesity-associated systemic inflammation persists for as much as 1 month, as indicated by IL-6 and CRP levels (549, 550, 620). Some of this inflammation has been attributed to the surgery itself (545). However, by 6 to 12 months post-surgery, circulating IL-6, CRP, and MCP-1 are typically reduced below pre-surgery levels (548, 549, 620–627), an effect that may be due to fat loss. Importantly, it is not yet clear what effect weight loss due to bariatric surgery has specifically on adipose tissue inflammation. Some studies have reported reduced levels of adipose tissue inflammation following 15–17% weight loss mediated by bariatric surgery (547, 548, 628, 629), while others have shown no changes in adipose tissue inflammation following 7–37% weight loss (549, 550, 630). With insulin sensitivity being substantially improved in all of these studies, these latter studies present a potential disconnect between adipose tissue inflammation and insulin sensitivity that requires further study. However, it must be noted that the adipose tissue sampled in these studies was from subcutaneous depots, due to ease of sampling. Given that visceral WAT is more prone to inflammatory changes, it is possible that visceral WAT inflammation is more impacted by bariatric surgery than subcutaneous WAT.

Bariatric surgery has been shown to upregulate FGF21 in humans, an effect that appears to be specific to RYGB-induced weight loss, as this effect is not observed following weight loss due to caloric restriction or sleeve gastrectomy (631–635). Importantly, it is not known if such FGF21 derives from the liver or adipose tissue. One study has shown that increased FGF21 is associated with improved HOMA-IR in RYGB subjects, an effect that remains when adjusted for adiposity (634), introducing the possibility that elevated FGF21 levels serve to impact glucose homeostasis. Given that FGF21 has been shown to be elevated in obesity, and in particular in subjects with insulin resistance (634), the notion that FGF21 levels would become even further elevated following RYGB surgery, a procedure which rapidly improves insulin sensitivity, represents a paradox. It has been proposed that obesity-associated increased FGF21 levels reflect a “spill-over” from cells that are experiencing metabolic stress (636); however, this hypothesis does not explain the further increased FGF21 levels that accompany RYGB.

Various forms of bariatric surgery have been shown to evoke long-term benefits including sustained and considerable weight loss as well as rapid and sustained remission of T2DM and reduced risk of CVD-related mortality (489). A recent meta-analysis has estimated that on average, patients exhibit a 48% reduction in macrovascular events with a 79% reduction in mortality more than 5 years following bariatric surgery (637). Similarly, long-term follow-up (>17 years) post-surgery in the Swedish Obesity Study showed a 32% reduction in macrovascular complications in T2DM subjects, with 29% fewer myocardial infarctions and a 29% decrease in stroke incidence (638, 639). Bariatric surgery also is associated with improved hypertension, but not a reduced risk of incident hypertension (640). Interestingly, the CRP reduction observed following bariatric surgery was most pronounced in subjects that regained the most insulin sensitivity (624), suggesting an important link between improved glucose metabolism and CVD.

### Thiazolidinediones (TZDs)

TZDs are synthetic peroxisome proliferator-activated receptor gamma (PPARγ) activators that have been used to treat T2DM for decades (641–643). The ability of TZDs to improve insulin resistance is clear; however, TZDs also promote adipogenesis and subsequent weight gain (644–647), and are thus not popular choices among patients who don't want to gain weight, even if it is “metabolically healthy” weight gain. The mechanism for such improvements in insulin sensitivity in the face of weight gain appears to be through the induction of adiponectin by TZDs (648), which has known insulin-sensitizing properties as described above.

Activation of PPARγ by TZDs not only enhances adipogenesis, it also alleviates inflammatory cytokine secretion associated with obesity (649) and reduces ectopic fat deposition in tissues such as the liver and skeletal muscle (650). There appears to be a reciprocal relationship between inflammatory cytokines and adiponectin. For example, *in vitro* experiments in cultured adipocytes revealed that treatment with adiponectin reduces cytokine secretion (651, 652), while treatment with cytokines drastically reduces adiponectin expression and secretion (648, 653, 654). Due to greater adipose lipid storage potential, TZDs should therefore reduce plasma triglyceride levels, which appears to be the case for pioglitazone but not rosiglitazone (655–657). This may in part account for the beneficial cardiovascular effects of pioglitazone in a clinical trial (658).

### Anti-Inflammatory Approaches

Characteristic features of MUHO and the metabolic syndrome include adipose tissue and systemic inflammation, which may play a role in the pathogenesis of atherosclerotic CVD. Therefore, an approach that inhibits inflammation would seem logical.

The CANTOS trial, in which CVD events were reduced using an IL-1β antagonist, canakinumab (659), was the first successful proof of concept study using an anti-inflammatory approach for the prevention of recurrent CVD events. A more recent study showed that colchicine, an old drug that has powerful anti-inflammatory properties, reduced recurrent ischemic events when administered after a myocardial infarction (660). Statins, which inhibit 3-hydroxy-3-methyl-glutaryl-coenzyme A reductase (HMG-CoA reductase) to reduce LDL cholesterol levels, also have anti-inflammatory properties (661–664). Whether this anti-inflammatory effect of statins plays a role in the well-documented effect of statins in inhibiting clinical CVD events and CVD mortality (665, 666) is unknown. Even less is known about the effect of statins on inhibiting inflammation in adipose tissue, although statins have been shown to reduce epicardial fat accumulation (667). A clue to the potential role of statins in adipose tissue inflammation is provided by the recent demonstration that myeloid-specific deletion of HMG-CoA reductase improved glucose tolerance in obesity induced by a high fat diet, as a result of decreased macrophage recruitment into adipose tissue (668). These changes occurred independently of weight loss and provide impetus for further studies on the effect of statins on adipose tissue inflammation. Regardless, the effect of statins on adipose tissue inflammation is an area that warrants further investigation.

### Modulating the Gut Microbiota

The trillions of bacteria that reside within our digestive tract, termed gut microbiota, play an important symbiotic role in shaping our metabolic health. The specific bacterial populations that inhabit our gut can have substantial metabolic impact in relation to obesity, as it is becoming increasingly recognized that that the gut microbiota may contribute to the pathology of obesity (669–671). Dysbiosis, or microbial imbalance in the body, has been associated with obesity in both humans and mice, and can be reversed with weight loss (672–675). Germ-free mice that do not possess gut microbiota are protected from diet-induced obesity and insulin resistance (676, 677), and the obesity phenotype can be conferred by transplantation of cecal contents from obese mice into lean germ-free mice (678), suggesting that the “obese microbiome” is sufficient to cause obesity. It is known that gut bacteria can influence distinct host organ systems indirectly and specifically through the release of particular microbial metabolites such as bile acids, short-chain fatty acids (SCFA), and others. Adipose tissue is a notable target of these microbial metabolites (679). As such, treatments that target the microbiome and modulate microbial metabolism could improve metabolic health.

There is growing evidence that gut dysbiosis can contribute directly to atherosclerotic CVD (669–671, 680). Gut microbial imbalance could modulate atherosclerosis by several mechanisms, including but not limited to: (1) promotion of metabolic endotoxemia due to decreased intestinal barrier integrity, leading to systemic inflammation; (2) altered cholesterol metabolism through the modification of bile acid metabolism, or (3) microbial production of specific beneficial or harmful metabolites with local and/or systemic activity, such as short-chain fatty acids (SCFA). These processes are described below.

#### Metabolic Endotoxemia

Gut dysbiosis has been associated with elevated intestinal permeability, or a “leaky gut” (681–683). Increased intestinal permeability allows inflammatory bacterial components to enter the systemic circulation to trigger an inflammatory response in diverse tissues such as the liver and adipose tissue. Obese mice and humans have been shown to exhibit gut dysbiosis (670), with increased proportions of endotoxin-producing gut bacteria and elevated circulating levels of lipopolysacharide that correlate with metabolic disease state such as obesity or T2DM (684, 685). Such metabolic endotoxemia is reduced following antibiotic treatment (681) or RYGB surgery-induced weight loss (627). Thus, a compromised intestinal barrier may contribute to systemic inflammation that is characteristic of obesity and CVD (686).

#### Bile Acid Metabolism

Gut dysbiosis contributes to dysregulated bile acid metabolism (687), leading to hyperlipidemia and hyperglycemia (688, 689). Bile acids produced by the liver facilitate the absorption of dietary fat in the small intestine, and are known to regulate lipid and glucose metabolism through the FXR (690, 691). FXR activation by bile acids initiates a negative feedback pathway, such that bile acid synthesis is inhibited when FXR is activated. Intestinal microbiota are capable of producing secondary bile acids from ~5 to 10% of hepatic-derived bile acids that affect host physiology by serving as FXR agonists, thus resulting in a smaller bile acid pool due to the inhibition of primary bile acid synthesis (692, 693). Subjects with obesity and/or T2DM have been shown to have fewer plasma secondary bile acids in comparison to healthy control subjects (694), an effect that may reflect the altered gut microbial composition observed in obesity. Adipocytes express the major G-protein-coupled bile acid receptor, TGR5, and FXR (695–697), and obesity is accompanied by reduced FXR expression in WAT (697); thus, bile acid signaling to adipose tissue could play a role in modulating adipose tissue inflammation and/or lipid metabolism (698). Secondary bile acids have been shown to exert an anti-inflammatory phenotype in macrophages and hepatocytes (699–701). Bariatric surgery increases plasma bile acid concentrations before any significant weight loss has been achieved (702–704). Metabolic benefits from bariatric surgery, including weight loss and improved glucose metabolism, were absent in mice lacking the TGR5 receptor (705), suggesting an important role for bile acids in the metabolic improvements associated with bariatric surgery. Indeed, adipocyte TGR5 is required for adipogenesis and a metabolically healthy adipokine profile, including secretion of adiponectin and repression of inflammatory cytokines (706, 707). Similarly, deficiency of FXR promotes adipocyte dysfunction, exemplified by impaired adipogenesis, defective insulin signaling, and reduced lipid storage capacity (697). Collectively, these previous studies suggest that intact bile acid signaling is required for adipocyte homeostasis. Thus, equilibrium between dietary-intestinal- and microbiome-intestinal-derived bile acids is important for metabolic health associated with lipid metabolism. The gut microbiota composition and metabolism are therefore important contributors to metabolic health.

#### SCFA Metabolism

SCFA, including predominantly acetate, propionate, and butyrate, are produced in the gut to varying degrees, depending on the fermentable carbohydrate-based substrates available (i.e., dietary fiber quantity and type) and the particular bacterial populations that are present (i.e., microbiota composition). SCFA serve as signaling molecules to remote organ systems, with impacts on autonomic regulation of systemic blood pressure, systemic inflammation, and other cellular functions. Dysbiotic gut bacteria that is observed in metabolic pathologies such as obesity and T2DM has been characterized by taxonomic shifts that produce fewer SCFA, with notably less butrate produced in the gut (708–710). Evidence from pre-clinical models suggests that SCFA administration could improve metabolic disease states such as obesity, T2DM, and atherosclerosis (711–714). Adipocytes express high levels of key receptors for SCFA, including GPR43 (715). Genetic deletion of GPR43 from adipocytes results in spontaneous obesity, while overexpression of adipocyte GPR43 protects mice from obesity (713). As such, adipose homeostasis can be directly modulated by the gut microbial composition and subsequent SCFA profile. Health benefits of giving SCFA to obese rodents include weight loss (712), improved glucose metabolism, reduced inflammation (716–719), and reduced LDL-cholesterol (720, 721), among others.

#### Gut Inflammation/Adipose Tissue Cross Talk

The gut microbiota are now considered to be a distinct organ system with endocrine properties that can directly and profoundly modulate the host immune system (722, 723). Under healthy conditions, the commensal (“normal”) gut microbiota play a prominent role in host homeostatic immunity, an essential function to limit the pathogenic potential of gut microbes, via innate and adaptive mechanisms (724, 725). When gut bacteria become dysbiotic, resulting immune deficiencies may contribute to the pathogenesis of obesity, T2DM, and CVD (726). The precise mechanisms by which gut microbiota modulate host immunity [reviewed in (709)] are beyond the scope of this review. However, some mechanisms by which microbial-derived metabolites can modulate adipose tissue function will be described herein. SCFA such as butyrate have been shown to dampen subcutaneous and visceral WAT inflammation by inhibiting NFκB activation (727, 728). Similarly, secondary bile acids negatively correlate with inflammatory pathways in WAT, suggesting an anti-inflammatory effect (729). Bacterial endotoxin, circulating levels of which increase during metabolic diseases that exhibit metabolic endotoxemia, readily promotes adipose tissue inflammation by activating toll-like receptor 4 (TLR4), which is highly expressed in adipocytes as well as macrophages (730). Thus, various metabolites produced by the gut microbiota are known to modulate adipose tissue inflammation directly through the circulation.

#### Probiotics, Prebiotics, and Synbiotics

Probiotics, prebiotics, and the combined synbiotics could provide an avenue for increased endogenous production of secondary bile acids and/or particular SCFA by modulating the composition of the gut microbiota. Probiotics are commercial preparations of live bacteria designed to be ingested, with the intention of colonizing the gut with the ingested bacteria, or at a minimum to confer a health benefit to the host. Prebiotics, on the other hand, are non-digestible dietary substrates designed to promote an abundance of gut-healthy bacteria, with inferred benefit to the host (731). Synbiotics are preparations that combine particular pre- and pro-biotics, as it is becoming clear that defined fiber substrates increase probiotic colonization efficiency. Pre- and pro-biotics and synbiotics are relatively inexpensive alternatives to conventional CVD medications, with fewer side effects (732). Mechanisms by which pre- and pro-biotic-mediated changes in the gut microbiota may improve adipose function are still emerging, but may include the promotion of an anti-inflammatory milieu (including reducing intestinal permeability to decrease circulating endotoxins), enhancing fat oxidation, recruitment of beige adipocytes, increased energy expenditure, and improved lipoprotein profile, which collectively could improve insulin sensitivity and reduce ectopic fat to combat T2DM and CVD (733–739). While it is generally accepted that particular pre- and pro-biotics reduce diet-induced weight and adiposity gain in animal models (736, 740–743), human intervention studies to date showing efficacy of probiotic treatment are still emerging (744, 745), warranting further study (746).

## Concluding Remarks

Obesity results in many changes to adipose tissue, including adipocyte hypertrophy and hyperplasia, infiltration of inflammatory cells, changes in the ECM, and altered adipokine secretion patterns. A critical determinant of whether obesity is likely to lead to metabolic complications such as insulin resistance, the metabolic syndrome, T2DM and CVD is the site where adiposity increases, particularly intra-abdominal, epicardial and perivascular depots, as well as other ectopic sites such as liver, skeletal muscle and pancreas. Ectopic fat accumulation at these sites demonstrate different metabolic, adipokine, and inflammatory profiles from excess white adipose tissue that accumulates subcutaneously, which is predominantly in a lower body distribution and contributes to a less unhealthy form of obesity. Several mechanisms by which these metabolic and inflammatory changes to different adipose tissue depots could influence the metabolic syndrome and its downstream consequences are potential targets for intervention. Various strategies for the treatment of T2DM and/or CVD, including lifestyle- and surgically-mediated weight loss as well as pharmacological or naturopathic methods, also have notable impacts on adipose tissue, which are important to consider.

## Author Contributions

LH and AC reviewed the literature and contributed to the preparation of this manuscript.

### Conflict of Interest

The authors declare that the research was conducted in the absence of any commercial or financial relationships that could be construed as a potential conflict of interest.

## Abbreviations

ALT: Alanine transaminase
M2: Alternatively activated macrophages
AMPK: AMP-activated protein kinase
ARG1: arginase-1
AST: aspartate transaminase
β-AR: beta-adrenergic receptor
BMI: body mass index
BAT: brown adipose tissue
CRP: C-reactive protein
CVD: cardiovascular disease
CT: computed tomography
DAGs: diacylglycerols
DEXA: dual-energy X-ray absorptiometry
eNOS: endothelial nitric oxide synthase
epiWAT: epicardial white adipose tissue
ECM: extracellular matrix
FXR: farnesoid X receptor
FGFs: fibroblast growth factors
FGF21: fibroblast growth factor 21
FDG: 2-[^18^F]fluoro-2-deoxyglucose
FFA: free fatty acid
TGR5: G-protein-coupled bile acid receptor
GLP-1: glucagon-like peptide-1
HDL: high-density lipoprotein
HMG-CoA reductase: 3-hydroxy-3-methyl-glutaryl-coenzyme A reductase
HOMA-IR: homeostatic model assessment of insulin resistance
HSL: hormone sensitive lipase
IKK: IkB kinase
IRS-1: insulin receptor substrate-1
ICAM-1: intercellular adhesion molecule-1
IFNγ: interferon γ
IRF3: interferon regulatory factor 3
IL-1Ra: interleukin-1 receptor antagonist
IL-6: interleukin-6
JNK: c-Jun N-terminal kinase
LEPR: leptin receptor
LXR: liver X receptor
LDL: low-density lipoprotein
MGL1: macrophage galactose type C-type Lectin/CD301a/CLEC10A
MHC: major histocompatibility complex
CD206: mannose receptor
MWAT: mesenteric white adipose tissue
MMe: metabolically activated macrophage
MHO: metabolically healthy obesity
MUHO: metabolically unhealthy obesity
MCP-1: monocyte chemotactic protein-1
NKT cells: natural killer T cells
NAFLD: non-alcoholic fatty liver disease
NE: norepinephrine
NFκB: nuclear factor kappa-B
OWAT: omental white adipose tissue
PVAT: perivascular adipose tissue
PPARα: peroxisome proliferator-activated receptor alpha
PPARγ: peroxisome proliferator-activated receptor gamma
RWAT: retroperitoneal white adipose tissue
SAA: serum amyloid A
SCFA: short-chain fatty acids
SGLT-2: sodium glucose co-transporter-2
SCD1: steroyl-CoA desaturase-1
SNS: sympathetic nervous system
TZDs: thiazolidinediones
TLR4: toll-like receptor 4
TNFα: tumor necrosis factor alpha
T2DM: type 2 diabetes mellitus
UCP-1: uncoupling protein-1
VCAM-1: vascular cell adhesion molecule-1
VEGF: vascular endothelial growth factor
VLDL: very low-density lipoprotein
WAT: white adipose tissue.
